# High-Density SNP Genotyping of Tomato (*Solanum lycopersicum* L.) Reveals Patterns of Genetic Variation Due to Breeding

**DOI:** 10.1371/journal.pone.0045520

**Published:** 2012-09-20

**Authors:** Sung-Chur Sim, Allen Van Deynze, Kevin Stoffel, David S. Douches, Daniel Zarka, Martin W. Ganal, Roger T. Chetelat, Samuel F. Hutton, John W. Scott, Randolph G. Gardner, Dilip R. Panthee, Martha Mutschler, James R. Myers, David M. Francis

**Affiliations:** 1 Department of Horticulture and Crop Science, The Ohio State University, Ohio Agricultural Research and Development Center, Wooster, Ohio, United States of America; 2 Seed Biotechnology Center, University of California Davis, Davis, California, United States of America; 3 Department of Crop and Soil Sciences, Michigan State University, East Lansing, Michigan, United States of America; 4 TraitGenetics GmbH, Gatersleben, Germany; 5 C. M. Rick Tomato Genetic Resource Center, University of California Davis, Davis, California, United States of America; 6 University of Florida, Gulf Coast Research and Education Center, Wimauma, Florida, United States of America; 7 Department of Horticultural Science, North Carolina State University, Mountain Horticultural Crops Research and Extension Center, Mills River, North Carolina, United States of America; 8 Department of Plant Breeding and Genetics, Cornell University, Ithaca, New York, United States of America; 9 Department of Horticulture, Oregon State University, Corvallis, Oregon, United States of America; University of New England, Australia

## Abstract

The effects of selection on genome variation were investigated and visualized in tomato using a high-density single nucleotide polymorphism (SNP) array. 7,720 SNPs were genotyped on a collection of 426 tomato accessions (410 inbreds and 16 hybrids) and over 97% of the markers were polymorphic in the entire collection. Principal component analysis (PCA) and pairwise estimates of *F*_st_ supported that the inbred accessions represented seven sub-populations including processing, large-fruited fresh market, large-fruited vintage, cultivated cherry, landrace, wild cherry, and *S. pimpinellifolium*. Further divisions were found within both the contemporary processing and fresh market sub-populations. These sub-populations showed higher levels of genetic diversity relative to the vintage sub-population. The array provided a large number of polymorphic SNP markers across each sub-population, ranging from 3,159 in the vintage accessions to 6,234 in the cultivated cherry accessions. Visualization of minor allele frequency revealed regions of the genome that distinguished three representative sub-populations of cultivated tomato (processing, fresh market, and vintage), particularly on chromosomes 2, 4, 5, 6, and 11. The PCA loadings and *F*_st_ outlier analysis between these three sub-populations identified a large number of candidate loci under positive selection on chromosomes 4, 5, and 11. The extent of linkage disequilibrium (LD) was examined within each chromosome for these sub-populations. LD decay varied between chromosomes and sub-populations, with large differences reflective of breeding history. For example, on chromosome 11, decay occurred over 0.8 cM for processing accessions and over 19.7 cM for fresh market accessions. The observed SNP variation and LD decay suggest that different patterns of genetic variation in cultivated tomato are due to introgression from wild species and selection for market specialization.

## Introduction

Selection of favorable alleles through domestication and breeding has led to dramatic changes in seed and fruit attributes, plant habit, and productivity. For tomato (*Solanum lycopersicum* L), breeding has involved the competing forces of narrowed genetic variation due to best by best crosses followed by selection [1], [2], and the expansion of genetic variation due to the introgression of genes for biotic stress resistance from wild species [3]–[5]. The long history of crossing to wild relatives has broadened the genetic diversity in contemporary germplasm relative to vintage and landrace germplasm [6]–[9]. In addition, breeding for distinct market classes and production systems has led to genetic differentiation in contemporary germplasm. For example, processing and field-grown fresh market tomatoes are now distinct sub-populations [8], [9].

Novel sequencing technologies have uncovered sufficient variation to investigate the effect of human selection across an entire plant genome [10]. Single nucleotide polymorphisms (SNPs) are a predominant form of sequence variation among individuals representing as much as 90% of the genetic variation in any species [11]. SNPs are distributed throughout a genome, they provide stable markers for genetic analysis, and their detection is amenable to automation. It is increasingly cost and time efficient to genotype large populations in a high-throughput manner. Because of these advantages, SNPs have become a marker system of choice for genetic analysis in plant species. In tomato, SNPs have been discovered using several methods: *in silico* mining of expressed sequence tag (EST) databases [12]–[14], intron and amplicon sequencing of conserved orthologous set (COS) genes [15], [16], and hybridization to oligonucleotide arrays [8]. Recently, 62,576 non-redundant SNPs were identified based on transcritpome sequences for six tomato accessions [10].

High-throughput SNP discovery has been accompanied by the development of array-based genotyping platforms that permit rapid scoring of several thousand markers in parallel [17]. Such SNP genotyping methods have facilitated high-density genetic map construction and genome-wide association analysis. For example, the use of a maize array with 49,585 SNPs produced two genetic linkage maps with 20,913 and 14,524 markers, respectively [18]. In tomato, we used the “SolCAP” array with 7,720 SNPs to generate high-density genetic maps using two F_2_ interspecific populations: 3,503 markers in the *S. lycopersicum* LA0925 x *S. pennellii* LA0714 (EXPEN 2000) population and 4,491 markers in the Moneymaker x *S. pimpinellifolium* LA0121 (EXPIM 2012) population [19]. An array with 44,100 SNPs was used to genotype 413 diverse accessions of rice and analyzed for association with 34 quantitative traits [20]. SNP data from such arrays can also be a resource for germplasm management in breeding programs and has a role in genomic selection strategies for crop improvement [18], [21].

The analysis of variation in tomato populations has often focused on differences between cultivated and wild species. In contrast, relatively little is known about which genes or genomic regions distinguish market classes within cultivated genepools. In the early 1900s, significant effort was placed on the evaluation of wild germplasm as a source of new resistance genes [3], [4]. Wide crosses were used to introduce new sources of resistance, with several varieties in commerce carrying introgressed genes by the late 1930s and early 1940s. These efforts often had a regional focus, and it remains unclear how introgressed regions are dispersed within breeding programs [22]. In addition, there was a concerted effort to breed for distinct plant habits and fruit characteristics for the processing and fresh market tomato industries. These efforts were initiated in the 1940s and resulted in the first cultivars suitable for machine harvest by the early 1960s [23]. At least three sources of *S. pimpinellifolium* were incorporated into processing breeding in order to develop compact plants amenable to “once over” (i.e. destructive) harvest [23].

In order to investigate genetic variation on the tomato genome due to contemporary breeding, we subjected a collection of 426 accessions to high-throughput genotyping using the SolCAP SNP array. The tomato accessions represented different market classes of cultivated tomato and closely related wild species. Knowledge of the genetic and physical organization of the SNPs [19] allowed us to conduct population level analysis based on the germplasm panel. SNP genotypes from the array were analyzed to identify sub-populations, and to assess genetic differentiation and diversity between and within sub-populations. We also investigated patterns of linkage disequilibrium (LD) within each chromosome in three sub-populations of large-fruited cultivated accessions (processing, fresh market, and vintage). The population level analysis revealed regions of the genome with high genetic variation between sub-populations, suggesting that historical breeding practices have led to different patterns of genetic variation in cultivated tomato germplasm.

## Results

### Array-based SNP Genotyping

We used an array consisting of 7,720 SNPs distributed throughout the genome [10], [19] to genotype 426 accessions (410 inbreds and 16 hybrids; referred to as the SolCAP germplasm) (Table S1 and Table S2). The hybrids were chosen to maximize heterozygosity and develop a cluster file for the GenomeStudio software (Illumina Inc., San Diego, CA, USA). In order to establish an accurate and automatic genotype calling procedure for the GenomeStudio software that is usable across the entire genepool of cultivated tomato, clustering based on the SolCAP germplasm was cross-validated with a cluster file based on 92 hybrids developed independently by TraitGenetics [19]. The resulting high-quality cluster file is available through the eXtension website [24].

In order to assess the quality of SNP calls, we duplicated 34 accessions that were randomly selected using independent DNA preparations and genotyping facilities. The average proportion of consistent calls across all accessions was 98.7%. Cluster analysis was performed, and for all 34 accessions, the nearest neighbor was the duplicate accession. The proportion of consistent calls increased to 99.3% by excluding three accessions, OH981136 (processing, 91.8%), Purple Clabash (vintage, 90.0%), and Principle Borghese (vintage, 94.1%). One of the duplicate samples for OH981136 showed higher rates for no call (8.8%) and heterozygote call (8.4%) relative to the other sample (0.7% for no call and 0.2% for heterozygote call). The data quality between two samples of Purple Clabash was also different from each other (0.7% vs. 5.7% for no call). Heterozygote call rates were similar for the two samples. The duplicate samples of Principe Borghese differed by 5.9%, but showed similar rates for no call (0.8% vs. 1.0%) and heterozygote call (0.2% vs. 0.2%). These results suggest that overall reproducibility is high, and that differential calls may result from DNA quality that affects the percentage of “no call”, residual heterozygosity, and variation within accessions.

The data were analyzed to determine the polymorphism rate based on the inbred accessions. A total of 7,500 SNPs (97.2%) were polymorphic and 61 SNPs (0.8%) were monomorphic (Table 1). Among the 7,500 polymorphic SNPs, there were 7,375 SNPs with <10% missing data, 84 SNPs with 10–20% missing data, and 41 SNPs with >20% missing data. The SNPs with a high frequency of missing data were randomly distributed across accessions. Polymorphism of 123 SNPs could not be unequivocally determined because of a large amount (≥10.0%) of missing data (34 SNPs) or because polymorphism detection was due to a single homozygous allele and only heterozygote calls for the alternate allele (89 SNPs). It is possible that the heterozygotes actually represent duplicated genes, or the alternate allele was simply not present as a homozygote. These 89 SNPs were also randomly distributed across the genome. In addition, 36 SNPs failed to produce a genotype call because of poor signals (Table 1).

**Table 1:** Polymorphism of 7,720 SNP markers based on 410 inbred tomato accessions in the SolCAP germplasm collection.

Class	No. of Marker	Percentage (%)
Polymorphic	7,500 (7,375^1^)	97.2 (95.5)
Monomorphic	61	0.8
Undetermined^2^	123	1.6
No call	36	0.4
Total	7,720	100.00

1 Number of SNPs with less than 10% missing data.

2 The class includes SNPs that were either monomorphic with ≥10% missing data (34 SNPs) or polymorphic due to only heterozygote calls (89 SNPs).

The proportion of heterozygous SNPs was assessed within cultivated tomato germplasm and *S. pimpinellifolium* accessions. The heterozygosity with all scorable markers (7,684 SNPs) was 0.02 for processing, 0.01 for large-fruited fresh market, 0.01 for large-fruited vintage, and 0.04 for *S. pimpinellifolium* accessions. Using only markers that were polymorphic within each sub-population (range 3,700– 6,022 polymorphic markers), the processing, fresh market, and vintage accessions showed heterozygosity levels of 0.02, 0.01, and 0.02, respectively. The heterozygosity for *S. pimpinellifolium* was 0.05.

### Genetic Differentiation between Sub-populations

**Figure 1:**
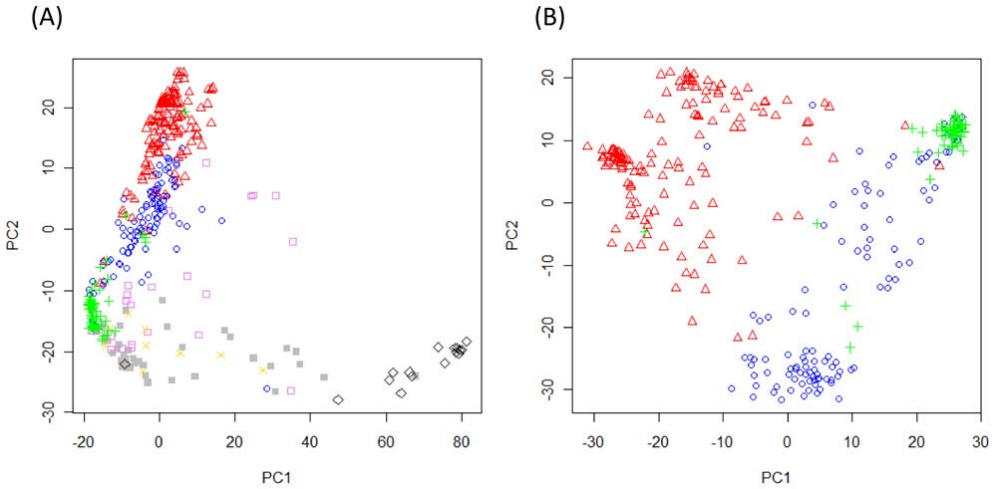
Principal component analysis (PCA) based on 4,393 SNP markers. The PCA was conducted separately using data for all sub-populations of the SolCAP germplasm (A) and data for only the three large-fruited cultivated sub-populations consisting of the processing, fresh market, and vintage accessions (B). The processing accessions are indicated Δ (red); fresh market, ○ (blue); vintage, + (green); cultivated cherry, □ (violet); landrace, × (gold); wild cherry, ▪ (gray); and *S. pimpinellifolium,* ◊ (black).

The 410 inbred accessions were first divided into five sub-populations based on *a priori* knowledge of germplasm pedigree, age, market class, and origin: 141 processing, 122 fresh market, 88 vintage, 43 wild cherry, and 16 *S. pimpinellifolium*. Principal component analysis (PCA) and linkage disequilibrium (LD) analysis supported separation of the fresh market sub-population into a group of 110 large-fruited accessions and 12 cherry accessions (cultivated cherry). Similarly, the vintage sub-population was divided into 61 large-fruited accessions, 15 cherry accessions which clustered with the cultivated cherry sub-population, and 12 landrace accessions (Figure 1 and Figure S1). This iterative analysis led to the definition of seven sub-populations for further analysis: 141 processing, 110 large-fruited fresh market (hereafter referred to as fresh market), 61 large-fruited vintage (hereafter referred to as vintage), 27 cultivated cherry, 12 landrace, 43 wild cherry, and 16 *S. pimpinellifolium*.

In the PCA analysis, the first two principal components (PC1 and PC2) explained 22% and 16% of the total variance, respectively. PC1 separated the *S. pimpinellifolium* accessions from all other sub-populations (*P*<0.001), while PC2 separated processing, fresh market, and vintage germplasm (*P*<0.01; Figure 1A). A subsequent PCA was conducted using only the processing, fresh market, and vintage sub-populations (Figure 1B). The second, more focused analysis validated the significant separation of the sub-populations. In addition, PCA based on only the cultivated accessions suggested further divisions within both processing and fresh market sub-populations (Figure 1B). Although most of the accessions were clustered into a corresponding sub-population, there were a few accessions that appear to be transitional and/or misclassified accessions. For example, Peto 460 (PI 600920; coordinates −22.1, −4.8 in Figure 1B) and Heinz 1370 (PI 341134; coordinates 4.6, −3.4 in Figure 1B) were grouped with the vintage accessions due to their date of release [25], but were developed as processing tomatoes. Similarly, Rio Grande (coordinates −12.5, 9.1 in Figure 1B) was classified as a fresh market accession, but clustered with the processing accession. This cultivar is an early “Roma” type tomato and was originally developed as a processing tomato. Clustering supports the processing origin of these three accessions (Figure 1B).

Pairwise analysis of *F*_st_ was used to test the significance of genetic differentiation between sub-populations (Table 2). In order to test effects of marker choice for this analysis, we estimated pairwise *F*_st_ using two independent subsets of SNP data derived from the array. Analysis was performed on all 410 inbred accessions for each marker set. In addition a re-sampling analysis was performed by randomly selecting n = 40 for processing, fresh market, and vintage sub-populations. Repetition of this analysis suggests that our conclusions are supported with balanced populations and across multiple marker subsets. Cultivated germplasm, including processing, fresh market, and vintage accessions were all significantly diverged (*F*_st_  = 0.29 to 0.41, *P*<0.005) (Table 2). The cultivated cherry, wild cherry, and landrace accessions were not differentiated from each other but were distinct from all other sub-populations (*F*_st_  = 0.13 to 0.64, *P*<0.005). The *S. pimpinellifolium* accessions were separated from all other sub-populations with estimates of pairwise *F*_st_ ranging between 0.57–0.81 (*P*<0.005; Table 2). The *F*_st_ analysis verified genetic differentiation between sub-populations defined by PCA.

**Table 2:** Pairwise estimates of *F*_st_ (θ) between sub-populations.

Sub-population	Proc		FM		Vintage	Cherry	Landrace	Wild cherry	Pimp
Processing (Proc)	0.00		0.29**		0.41**	0.27**	0.40**	0.38**	0.72**
Fresh market (FM)			0.00		0.27**	0.18**	0.28**	0.29**	0.72**
Vintage					0.00	0.13**	0.18**	0.20**	0.81**
Cultivated cherry (Cherry)						0.00	0.04^NS^	0.05*	0.58**
Landrace							0.00	0.04^NS^	0.64**
Wild cherry								0.00	0.57**
*S. pimpinellifolium* (Pimp)									0.00
Further division within sub-population	Proc 1	Proc 2	FM 1	FM 2	Vintage	Cherry	Landrace	Wild cherry	Pimp
Proc 1	0.00	0.27**	0.42**	0.40**	0.52**	0.34**	0.49**	0.44**	0.74**
Proc 2		0.00	0.52**	0.32**	0.47**	0.30**	0.45**	0.38**	0.75**
FM 1			0.00	0.32**	0.52**	0.34**	0.49**	0.42**	0.76**
FM 2				0.00	0.12**	0.10**	0.17**	0.19**	0.72**



Pairwise θ [92] was estimated using the Microsatellite analyzer v4.05 [93]. P-value was calculated based on 10,000 permutations with Bonferroni correction. NS, not significant,*P<0.05 and **P<0.005.

Further divisions within both processing and fresh market sub-populations detected in PCA were also tested by estimating pairwise *F*_st_. The processing accessions can be divided into two groups consisting of 82 and 52 accessions, respectively (Table S3). Seven accessions were not grouped because they were outliers, these tended to be CULBPT accessions which derive from recent crosses to fresh-market material. The two main processing groups were significantly differentiated from each other (*F*_st_  = 0.27 *P*<0.005) and distinct from the other germplasm (Table 2). Two groups of the fresh market tomatoes including 61 and 49 accessions each showed significant differentiation between them and from the other sub-populations (*F*_st_  = 0.10 to 0.76, *P*<0.005; Table 2).

### Levels of Polymorphism within Sub-populations

The level of genetic diversity within each sub-population was measured using allelic richness (*A*), expected heterozygosity (*He*), and polymorphic information content (PIC). Within cultivated germplasm, the highest estimates of *A*, *He*, and PIC were found in the cultivated cherry accessions (Table 3). Among the remaining cultivated sub-populations, the fresh market accessions showed higher *A*, while the landraces had the highest *He* and PIC values. The sub-population of vintage accessions contained the lowest variation for all three descriptors (Table 3). The further division of accessions within both processing and fresh market sub-populations showed higher estimates of these descriptive statistics relative to the vintage sub-population (Table 3).

**Table 3:** Descriptive statistics for genetic diversity within sub-populations.

Sub-population^1^	Sample size	*A* 2	*He* 3	PIC^4^
Processing (Proc)	141	1.49	0.16	0.13
Proc 1	82	1.47	0.13	0.11
Proc 2	52	1.38	0.12	0.09
Fresh market (FM)	110	1.59	0.16	0.13
FM 1	61	1.46	0.11	0.09
FM 2	49	1.61	0.16	0.13
Vintage	61	1.37	0.09	0.07
Cultivated cherry	27	1.89	0.27	0.21
Landrace	12	1.54	0.19	0.14
Wild cherry	43	1.89	0.26	0.21
*S. pimpinellifolium*	16	1.87	0.28	0.21

1 The further divisions within both processing and fresh market sub-populations are indicated with a number followed by each sub-population name (e.g. Proc 1 and Proc 2). Seven processing accessions were excluded for this grouping because they were outliners.

2 Allelic richness [94], [95].

3 Expected heterozygosity corrected for sample size [96].

4 Polymporphic Information Content [97].

The highest number of polymorphic markers (6,234 SNPs) was identified in the cultivated cherry sub-population. There were 5,909 and 5,650 polymorphic markers in the wild cherry and *S. pimpinellifolium* sub-populations (Table 4). For the contemporary accessions, 4,648 and 6,022 markers were polymorphic in the processing and fresh market sub-populations, respectively (Table 4). Fewer polymorphic markers were found in the vintage (3,700 SNPs) and landrace (3,159 SNPs) sub-populations. Distribution patterns of polymorphic SNP markers across 12 chromosomes were different between sub-populations (Table 4). For example, chromosome 8 showed proportionally lower SNP numbers for the processing and cultivated cherry accessions, as did chromosome 12 for the large-fruited fresh market and wild cherry tomatoes. Chromosome 10 had lower polymorphism rates for the vintage and landrace groups compared to contemporary processing and large-fruited fresh market sub-populations.

**Table 4:** Distribution of polymorphic SNP markers in seven sub-populations of the SolCAP germplasm.

	Processing		Fresh market		Vintage		Cultivated cherry		Landrace^1^		Wild cherry		*S. pimpinellifolium*	
Chr	SNP No.	%	SNP No.	%	SNP No.	%	SNP No.	%	SNP No.	%	SNP No.	%	SNP No.	%
1	266	5.7	345	5.7	210	5.7	369	5.9	232	7.3	446	7.5	370	6.5
2	456	9.8	764	12.7	336	9.1	664	10.7	352	11.1	763	12.9	689	12.2
3	450	9.7	497	8.3	321	8.7	543	8.7	340	10.8	564	9.5	486	8.6
4	707	15.2	751	12.5	574	15.5	789	12.7	422	13.4	672	11.4	674	11.9
5	666	14.3	634	10.5	520	14.1	684	11.0	207	6.6	499	8.4	617	10.9
6	436	9.4	602	10.0	215	5.8	638	10.2	248	7.9	417	7.1	406	7.2
7	159	3.4	307	5.1	188	5.1	305	4.9	228	7.2	338	5.7	353	6.2
8	147	3.2	292	4.8	146	3.9	255	4.1	198	6.3	326	5.5	280	5.0
9	209	4.5	403	6.7	163	4.4	419	6.7	164	5.2	348	5.9	306	5.4
10	159	3.4	332	5.5	120	3.2	337	5.4	126	4.0	363	6.1	312	5.5
11	785	16.9	791	13.1	740	20.0	904	14.5	395	12.5	864	14.6	869	15.4
12	203	4.4	290	4.8	160	4.3	317	5.1	241	7.6	298	5.0	279	4.9
unknown	5	0.1	14	0.2	7	0.2	10	0.2	6	0.2	11	0.2	9	0.2
Total	4,648	100.0	6,022	100.0	3,700	100.0	6,234	100.0	3,159	100.0	5,909	100.0	5,650	100.0

1 Latin American Cultivar.

**Figure 2:**
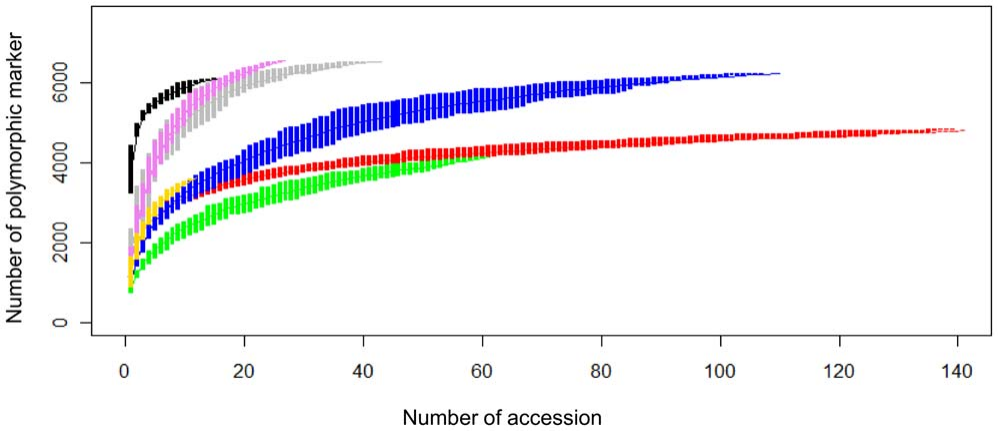
Rarefaction analysis to estimate the number of polymorphic markers in each sub-population. Accessions are coded as in Figure 1 with processing indicated by red; fresh market by blue; vintage by green; cultivated cherry, violet; landrace, gold; wild cherry, gray; and *S. pimpinellifolium,* black. Curves are plotted with standard deviations indicated by vertical bars.

The number of polymorphic markers in sub-populations increases with the size of the population. This fact led to concerns that our estimates of diversity might be skewed by population size differences despite the fact that *A* and *He* are adjusted for population size. To address the concern, we performed rarefaction analysis to estimate polymorphic marker accumulation curves [26]. For all seven sub-populations, the curves reached an asymptote within the population size sampled (Figure 2). The *S. pimpinellifolium*, wild cherry, and cultivated cherry sub-populations were the most diverse groups based on the curves. Within the large-fruited cultivated sub-populations, fresh-market accessions were more diverse than vintage accessions. The accumulation curves for the processing and vintage accessions merged at n = 60, despite the fact that they were well separated between n = 10 to 25. This result suggests that differences in number of polymorphisms detected between these sub-populations may be population size dependent.

### Minor Allele Frequency of SNP Markers

**Figure 3:**
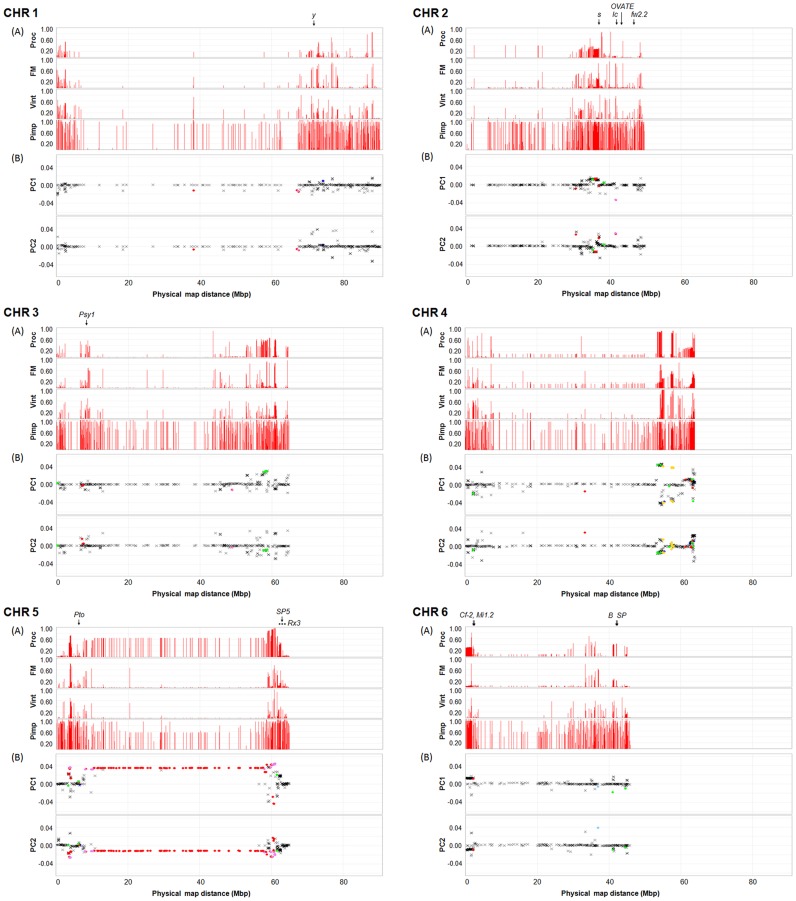
Minor allele frequency (MAF) patterns, PCA loadings, and *F*_st_ outliers on chromosomes 1 to 6. The minor allele was determined relative to allele calls for all 410 inbred accessions based on 7,310 SNPs, and then MAF was estimated and graphed for processing (Proc), fresh market (FM), and vintage (Vint) and *S. pimpinellifolium* (Pimp) sub-populations (A). The 28 tomato genes listed in Table S9 are positioned based on their coding sequences (arrow) and flanking markers (dotted line). The Y-axis represents allele frequency and the X-axis represents physical positions of the SNPs oriented with respect to the tomato genome sequence [102]. PCA loadings for PC 1 and PC 2 are graphed with candidates for loci under positive selection based on *F*_st_ outlier analysis [27], [28] (B). The candidate loci are indicated by dots (•) with a color scheme indicating pairwise comparisons that were significant: red for Proc vs. FM; green for Vint vs. Proc; blue for Vint vs. FM; violet for Proc vs. FM and Vint; sky blue for FM vs. Proc and Vint; and gold for Vint vs. Proc and FM. All other loci are indicated by X (black).

**Figure 4:**
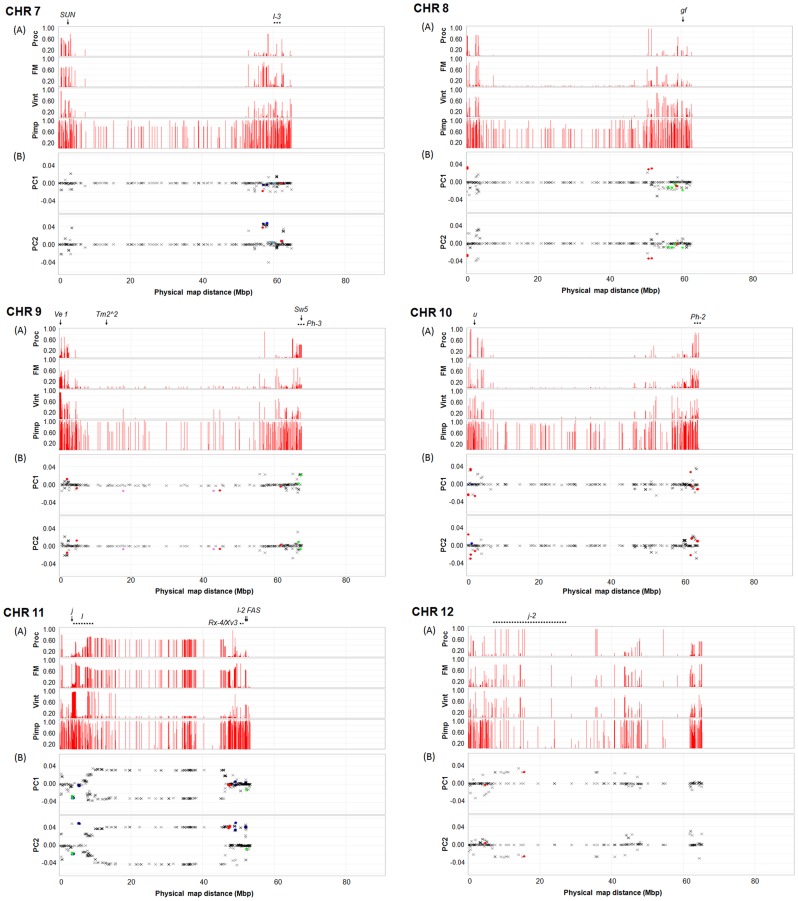
Minor allele frequency (MAF) patterns, PCA loadings, and *F*_st_ outliers on chromosomes 7 to 12. The minor allele was determined relative to allele calls for all 410 inbred accessions based on 7,310 SNPs, and then MAF was estimated and graphed for processing (Proc), fresh market (FM), and vintage (Vint) and *S. pimpinellifolium* (Pimp) sub-populations (A). The 28 tomato genes listed in Table S9 are positioned based on their coding sequences (arrow) and flanking markers (dotted line). The Y-axis represents allele frequency and the X-axis represents physical positions of the SNPs oriented with respect to the tomato genome sequence [102]. PCA loadings for PC 1 and PC 2 are graphed with candidates for loci under positive selection based on *F*_st_ outlier analysis [27], [28] (B). The candidate loci are indicated by dots (•) with a color scheme indicating pairwise comparisons that were significant: red for Proc vs. FM; green for Vint vs. Proc; blue for Vint vs. FM; violet for Proc vs. FM and Vint; sky blue for FM vs. Proc and Vint; and gold for Vint vs. Proc and FM. All other loci are indicated by X (black).

Minor alleles for 7,310 SNP markers with physical map positions were determined based on genotypic data of 410 inbred accessions. Minor allele frequency (MAF) was then estimated within each sub-population (Table S4). We graphed MAF in order to visualize genetic variation between three representative sub-populations of cultivated tomatoes (processing, fresh market, and vintage) along with *S. pimpinellifolium* accessions. These plots revealed different MAF patterns over the entire genome with chromosomes 2, 4, 5, 6, and 11 being particularly variable between the cultivated sub-populations (Figure 3A and Figure 4A). The processing and fresh market sub-populations showed unique MAF patterns on these chromosomes relative to the vintage sub-population. MAF patterns on chromosome 5 distinguished the processing sub-population from the fresh market sub-population (Figure 3A and Figure 4A). Common alleles in the *S. pimpinellifolium* accessions tended to be minor alleles in the cultivated sub-populations (Figure 3A and Figure 4A). Since the processing and fresh market sub-populations were further divided into two sub-groups, respectively, MAF was estimated within each sub-group and graphed (Table S4 and Figure S2). The processing sub-groups were distinguished by unique MAF patterns on chromosomes 5 and 11. The most variable MAF patterns between the large-fruited fresh market sub-groups were found on chromosome 11 (Figure S2).

### Loci Explaining Variation within Germplasm

PCA loadings measure the correlation between PC and SNP, and provide an estimate of how much each SNP contributes to variance. We extracted loadings from the PCA of only cultivated germplasm (Figure 1B) and displayed these values relative to physical position in order to visualize SNPs that contribute most to variance in the germplasm panel. There were 778 SNPs with absolute values of >0.02 for PC1. Most of these SNPs were found on chromosomes 4 (24.0%), 5 (32.6%), and 11 (31.1%). For PC2, 709 SNPs with absolute values of >0.02 were found on chromosomes 5 (12.3%) and 11 (52.9%) (Figure 3B, Figure 4B, and Table S5).

We also investigated loci that may be under positive selection between the three cultivated sub-populations using an *F*_st_ outlier method based the expected distribution of *F*_st_ and *He* [27], [28]. We identified 339 candidates for loci under positive selection between processing and fresh market as falling outside of the 95% confidence interval (Figure 3B, Figure 4B, and Table S6). A high portion of these loci (61.4%) were derived from chromosome 5, while 0.6–10.0% of the SNPs were distributed across the other 11 chromosomes. Comparison between processing and vintage germplasm detected 128 candidates for loci under positive selection (Figure 3B, Figure 4B, and Table S7). Among these, 57 loci (44.5%) were located on chromosome 4 and 35 loci (27.3%) on chromosome 5. For comparison between fresh market and vintage sub-germplasm, 208 loci were outliers based on a 95% confidence interval (Figure 3B, Figure 4B, and Table S8). Most of the loci were derived from chromosome 4 (42.6%) and chromosome 11 (43.5%). For all three pairwise comparisons, the candidate loci under positive selection were not randomly distributed within a chromosome.

To visualize the position of candidate genes that may have been selected for during breeding, we superimposed the position of 28 loci onto the physical map of MAF pattern, PCA loading, and *F*_st_ outlier detection. Candidate loci included genes that affect fruit size, shape, and color, disease resistance, and plant morphology (Table S9). Three genes for fruit shape and size, *fw2.2*, *OVATE*, and *lc* are located on chromosome 2 [29]–[32]. Of these genes, only *lc* appears to be a candidate for a locus under selection based on MAF patterns, PCA loadings, and *F*_st_ outlier detection of linked SNPs (Figure 3). The *OVATE* locus is polymorphic within all cultivated sub-populations, and therefore SNPs lack power to discriminate populations. The large fruited allele of *fw2.2* is fixed in the cultivated sub-populations. The *SUN* mutation which controls elongated fruit shape is found on chromosome 7 [33] and is present at a low frequency in both processing and vintage accessions (Figure 4). The genomic region for the *SUN* mutation contains some SNPs with high absolute values for PCA loadings, though none of these were detected in the *F*_st_ outlier analysis. Another fruit shape gene, *FAS* is found in a region of chromosome 11 [34] with high loadings for PC2, and LD between *FAS* and SNPs in this region may be responsible for detection of *F*_st_ outliers between processing (which lack *FAS*) and vintage (which are polymorphic for *FAS*) germplasm (Figure 4). Phenotypic data are available in flat file format through the SolCAP website [35] and in searchable form through the Sol Genome Network (SGN) ontology database using advanced search options with the stock number prefix, SCT; stock type, accession; stock editors, SolCAP project; and the organism as either *Solanum lycopersicum* or *S. pimpinellifolium* [36]. A detailed analysis of phenotypic data is provided in a separate publication [37].

Fruit color genes affecting skin and flesh color are distributed on chromosomes 1 (*y*), 3 (*Psy1*), 6 (*B*), 8 (*gf*), and 10 (*u*) [38]–[43]. Although *y*, *Psy1* and *gf* are found in regions of the genome with SNPs that have moderately high absolute values associated with PCA loadings and somewhat variable MAF between sub-populations, these regions do not appear to be highly discriminatory (Figure 3 and Figure 4). Regions on chromosome 1 and 3 were also not coincident with loci identified by the *F*_st_ outlier approach. The *old gold crimson* (*og^c^*) allele of the *B* gene encodes a mutation in the fruit specific lycopene beta-cyclase [41] and is segregating in all three cultivated populations. Thus minor variation in MAF patterns, PCA loadings, and the detection of a SNP under selection between processing and vintage are more likely a reflection of the closely linked *SP* allele. In contrast, the region of chromosome 8 containing *gf* contains several SNPs detected as outliers based on *F*_st_ between processing and vintage germplasm (Figure 4), which is consistent with the absence of *gf* mutations in processing populations and the presence of the mutant alleles in several vintage accessions. The allele of *u* gene for uniform ripening appears to fall in a region that is fixed in the processing germplasm. This allele is present at a high frequency in fresh market germplasm and at a low frequency in vintage germplasm (Figure 4). The region contains several SNPs with high absolute values for PCA loadings and also SNPs detected as *F*_st_ outliers.

The signals of genetic differentiation on chromosomes 5, 6, and 11 might be due to allelic variation in disease resistance genes, and reflect different introgression histories. Two resistance genes to bacterial disease, *Pto* and *Rx3* on chromosome 5 [44], [45] appears to be candidates for loci under selection based on MAF patterns, PCA loading, and *F*_st_ outlier detection (Figure 3). The region of chromosome 6 containing *Cf-2* and *Mi1.2* genes [46], [47] shows signals of selection distinguishing processing accessions from fresh market and vintage accessions (Figure 3). In general, *Cf* genes are deployed more frequently in fresh market material while *Mi* has been widely used in processing germplasm in California, but rarely used in processing accessions in the Midwestern U.S. Chromosome 11 contains one of the oldest introgressions, *I* for *Fusarium* resistance [48], [49]. This gene falls in a region of the genome that distinguishes contemporary germplasm from vintage germplasm based on MAF patterns (Figure 4). The region also contains some SNPs with high PC loadings. SNPs were detected as outliers at the 95% confidence level between processing and vintage germplasm. The region of chromosome 11 containing *Rx-4/Xv3* and *I-2* [50], [51] appears to be discriminatory between the cultivated sub-populations based on MAF patterns, PC2 loadings, and outlier SNPs (Figure 4). Other regions with signals for loci under selection include chromosomes 7 (*I-3*) [48], [52], chromosome 9 (*Ve1*, *Tm2* ^∧^ *2*, *Sw5*, and *Ph-3*) [53]–[57], and chromosome 10 (*Ph-2*) [58] (Figure 4). The genomic regions for *Ph-2* and *Ph-3* show MAF patterns that distinguish processing from fresh market and vintage sub-populations (Figure 4). SNPs were also detected in the regions by PCA loadings and *F*_st_ outlier analysis. These resistance genes have been deployed in several processing tomatoes [59]. The region of the genome containing *Tm2* ^∧^ *2* distinguishes fresh-market from vintage and processing accessions based on the presence of SNPs with a MAF of 10–15%, consistent with deployment of this resistance in fresh market accessions (Figure 4). However, this region was not detected based on PC loadings or *F*_st_ outlier detection, possibly due to low allele frequencies for markers in linkage disequilibrium with *Tm2* ^∧^ *2*.

Genes affecting plant morphology are distributed across several chromosomes. A region on chromosome 2 that appears to differentiate sub-populations contains the compound inflorescence gene (*s*) [60] (Figure 3). Chromosomes 5 and 6 contain genes (*SP5* and *SP*, respectively) that control plant habit and may be key selection points in differentiating vintage from contemporary sub-germplasm [61] and processing from fresh market [62], [63]. The *SP5* gene is in a region of the genome containing SNPs with high loadings for PC2, but does not appear to have been detected by *F*_st_ outlier analysis (Figure 3). The *jointless* gene (*j*) on chromosome 11 [64] falls in a region containing SNPs with high absolute values for PCA loadings and outlier SNPs (Figure 4). This region distinguishes processing from fresh market and vintage accessions, reflecting the near fixation of jointless accessions in this germplasm category. The genomic region for the *j-2* gene on chromosome 12 [65] contains SNPs with high absolute values for PCA loadings (Figure 4).

### Linkage Disequilibrium (LD) Analysis

**Figure 5:**
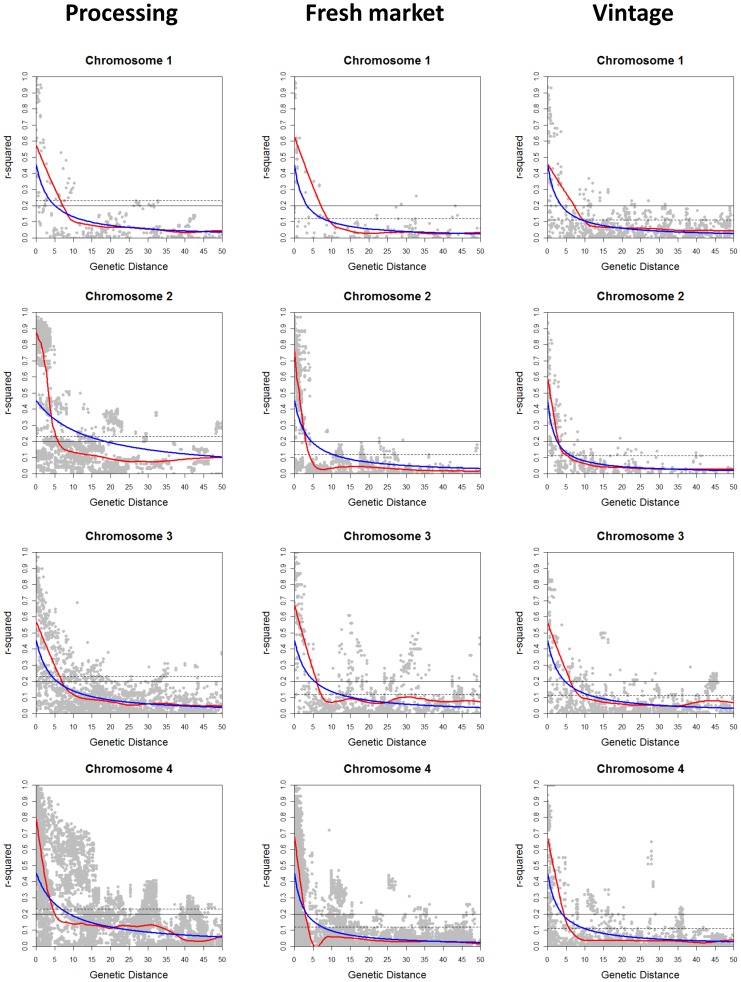
Linkage disequilibrium (LD) decay on chromosomes 1 to 4. LD measures *r^2^* against genetic map distance between pairs of SNP markers within each chromosome for processing, fresh market, and vintage sub-populations. Decay curves of locally weighted scatterplot smoothing (LOESS) [66] are represented by red, and decay curves of non-linear regression (NLR) [67] are represented by blue. Horizontal dashed and solid lines indicate the baseline *r^2^*values estimated using the 95^th^ percentile method (0.23 for processing; 0.12 for fresh market; and 0.11 for vintage) and a fixed *r^2^*value of 0.2, respectively.

**Figure 6:**
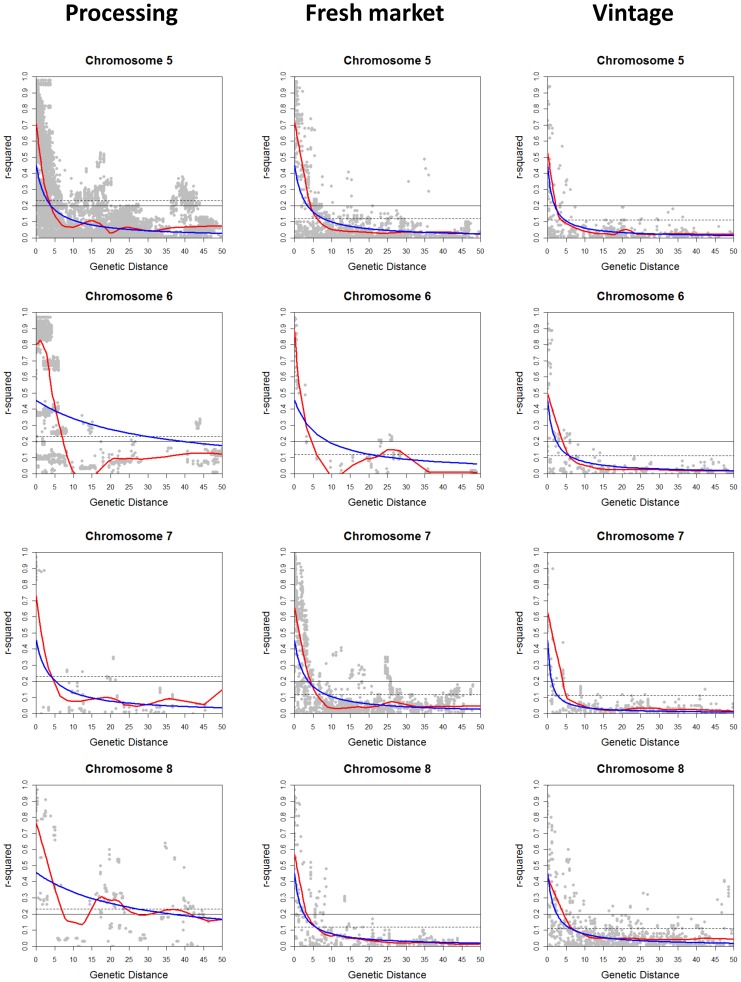
Linkage disequilibrium (LD) decay on chromosomes 5 to 8. LD measures *r^2^* against genetic map distance between pairs of SNP markers within each chromosome for processing, fresh market, and vintage sub-populations. Decay curves of locally weighted scatterplot smoothing (LOESS) [66] are represented by red, and decay curves of non-linear regression (NLR) [67] are represented by blue. Horizontal dashed and solid lines indicate the baseline *r^2^*values estimated using the 95^th^ percentile method (0.23 for processing; 0.12 for fresh market; and 0.11 for vintage) and a fixed *r^2^*value of 0.2, respectively.

**Figure 7:**
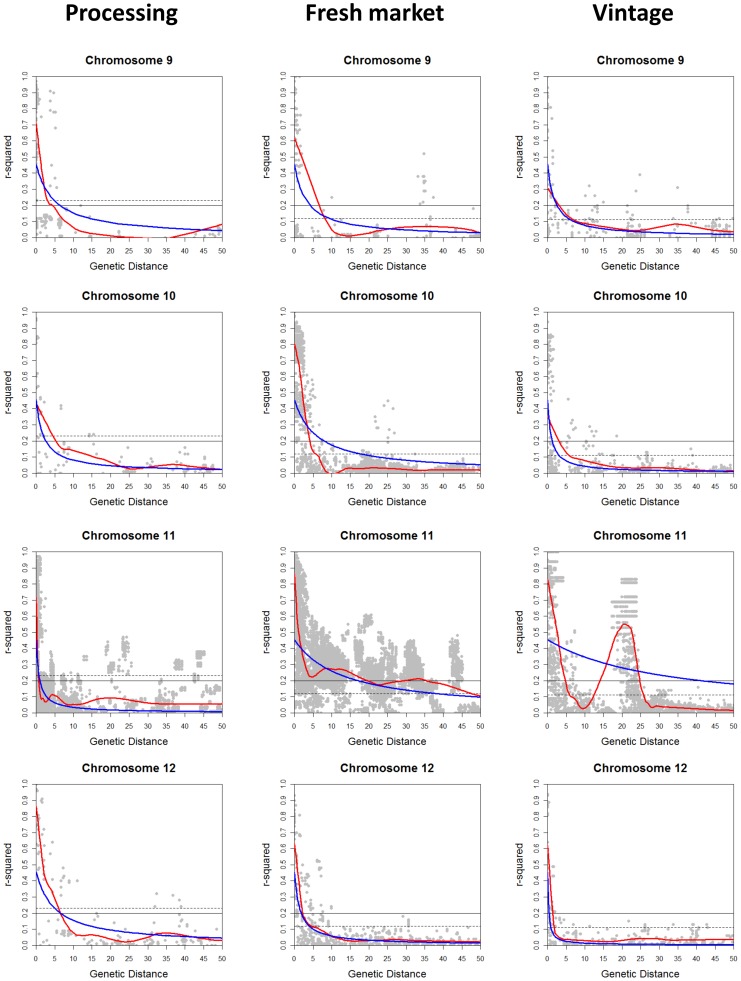
Linkage disequilibrium (LD) decay on chromosomes 9 to 12. LD measures *r^2^* against genetic map distance between pairs of SNP markers within each chromosome for processing, fresh market, and vintage sub-populations. Decay curves of locally weighted scatterplot smoothing (LOESS) [66] are represented by red, and decay curves of non-linear regression (NLR) [67] are represented by blue. Horizontal dashed and solid lines indicate the baseline *r^2^*values estimated using the 95^th^ percentile method (0.23 for processing; 0.12 for fresh market; and 0.11 for vintage) and a fixed *r^2^*value of 0.2, respectively.

The extent of LD across each chromosome was analyzed for the processing, large-fruited fresh market, and large-fruited vintage sub-populations. Pairwise *r^2^* was calculated using 1,572 polymorphic SNP markers with MAF of ≥0.1 for processing, 1,504 for fresh market, and 700 for vintage accessions. The *r^2^* values were plotted against the genetic distance, and curves of LD decay were fitted using locally weighted scatterplot smoothing (LOESS) [66] and non-linear regression (NLR) [67]. The LOESS and NLR methods estimated similar LD decay on 9 chromosomes for processing, 8 chromosomes for fresh market, and 11 chromosomes for vintage germplasm. In these chromosomes, the observed difference of the LD decay between the methods ranged from 0 to 4.9 cM (Table 5 and Figures 5, 6, 7). Over 10 cM difference for LD decay estimated by LOESS and NLR was found on chromosome 6 (28.1 cM for processing and 14.1 cM for fresh market); chromosome 8 (20.2 cM for processing); and chromosome 11 (9.6 cM for fresh market and >40 cM for vintage) (Table 5 and Figures 5, 6, 7). The use of either a fixed *r^2^* value of 0.2 or a value estimated using the 95^th^ percentile method resulted in similar values for LD decay over chromosomes in the three sub-populations.

**Table 5:** Chromosome by chromosome linkage disequilibrium (LD) analysis within three representative sub-populations of cultivated tomato.

	LD decay (cM)											
	95th percentile method^1^						Fixed method (*r* 2 = 0.2)					
	Processing		Fresh market		Vintage		Processing		Fresh market		Vintage	
Chr	LOESS^2^	NLR^3^	LOESS	NLR	LOESS	NLR	LOESS	NLR	LOESS	NLR	LOESS	NLR
1	6.6	4.1	9.3	7.7	9.9	9.4	7.2	5.3	7.7	3.6	7.2	3.8
2	5.4	14.2	4.1	10.2	5.8	6.6	6.0	18.7	3.3	4.6	3.0	2.7
3	6.3	4.4	7.2	12.1	8.6	11.8	6.9	5.7	6.1	5.4	6.1	4.7
4	4.6	6.9	3.8	7.4	5.8	9.9	5.2	9.0	2.9	3.5	4.4	4.1
5	3.5	3.1	6.1	7.7	3.8	4.7	3.9	4.1	4.3	3.5	2.5	1.9
6	7.1	35.2	6.0	20.1	5.8	6.0	7.4	40.6	5.0	9.7	4.1	2.8
7	4.0	4.0	6.0	8.3	4.9	2.8	5.0	6.4	4.4	3.8	4.1	1.1
8	7.7	27.9	5.7	5.7	6.7	6.3	7.7	36.7	3.3	2.8	4.4	2.5
9	3.5	5.0	9.0	9.5	7.8	6.5	4.7	6.6	6.9	4.7	4.1	2.7
10	5.2	2.2	5.8	17.7	5.8	3.6	6.0	2.8	4.2	8.0	3.6	1.4
11	0.8	0.8	47.6	38.0	6.0	n.d.	0.8	1.1	19.7	17.2	4.9	41.3
12	6.7	5.5	4.6	4.1	1.9	1.1	6.7	6.7	2.2	1.9	1.6	0.5
Average	5.0	9.8	10.1	13.1	6.4	6.8	5.5	12.5	6.2	6.1	4.4	6.3

1 The estimate of the 95th percentile baseline r2 value in each germplasm group was 0.23 in the processing varieties, 0.12 in the fresh market varieties, and 0.11 in the vintage varieties.

2 Logally weighted scatterplot smoothing [66].

3 Non-linear regression [67]. For NLR, the expected r2 was calculated using the model of Hill and Weir (1988).

n.d.  =  not determined.

Different patterns of LD decay were observed between chromosomes and sub-populations (Table 5 and Figures 5, 6, 7). Baseline *r^2^* values estimated using the 95^th^ percentile method ranged from 0.11 to 0.23. In the processing sub-population, the baseline *r^2^* value based on the 95^th^ percentile method was 0.23 and led to estimates of LD decay that ranged from 0.8 cM on chromosome 11 (both LOESS and NLR) to 7.7 cM on chromosome 8 (LOESS) and 35.2 cM on chromosome 6 (NLR) (Table 5 and Figures 5, 6, 7). LD decay estimated from the 95^th^ percentile baseline *r^2^* value of 0.12 in the fresh market sub-population ranged from 3.8 cM on chromosome 4 to 47.6 cM on chromosome 11 for LOESS, and ranged from 4.1 cM on chromosome 12 to 38 cM on chromosome 11 using NLR. With the 95^th^ percentile baseline *r^2^* value of 0.11 in the vintage sub-population, LD decay occurred over the shortest distance on chromosome 12 (1.9 cM for LOESS and 1.1 cM for NLR), while the greatest distance for LD decay was found on chromosome 1 (9.9 cM for LOESS) and chromosome 3 (11.8 cM for NLR) (Table 5 and Figures 5, 6, 7). Estimates of LD decay vary more on a chromosome to chromosome basis than those based on the method used to establish the LD cut-off or decay curve.

## Discussion

A high density array with 7,720 SNP markers was used to genotype the SolCAP germplasm panel consisting of 410 inbred accessions. This diverse germplasm enabled the development of a cluster file for accurate SNP calling with the GenomeStudio software (Illumina Inc. San Diego, CA, USA). Over 98% of SNP markers generated consistent calls between duplicate samples across 34 accessions, with differences due mostly to DNA quality. Also, 7,375 SNP markers (96%) were polymorphic with <10% missing data in the entire germplasm. We found that the level of heterozygosity (proportion of heterozygotes) was low, but variable between market classes of germplasm.

The SNPs provide excellent genome coverage, but their distribution is not reflective of chromosome size. For example, chromosome 1 is cytologically one of the largest, yet is underrepresented by SNPs relative to chromosome 11 which is cytologically small. It is possible that this distribution represents a distortion of the true measure of polymorphism, heterozygosity, or genetic diversity due to the sampling of markers. However, the germplasm presented in this study were well represented in the sequencing and SNP discovery pipeline [10] with five of the seven sub-populations contributing to sequenced germplasm. We found more variation in the level of polymorphism between chromosomes within a sub-population relative to the variation between sub-populations. These results suggest that there is little ascertainment bias within the red-fruited species, and that the chromosome to chromosome variation reflects breeding history rather than polymorphism discovery. The possibility of ascertainment bias when applied to more distant germplasm exists, and will be the subject of a future study.

Cultivated germplasm was divided into distinct sub-populations. The genetic differentiation between processing and fresh market germplasm reflects human selection for distinct ideotypes tailored to the needs of specific production systems. The contemporary processing and fresh-market germplasm were also distinct from vintage germplasm, which is consistent with previous findings [6]–[9]. The further division within processing germplasm confirms an earlier analysis based on the Bayesian model implemented in STRUCTURE [68] which demonstrated sub-structure consistent with breeding history and environmental adaptation [9]. We have not seen evidence for sub-structure within fresh market germplasm previously [9]. Upon close inspection, the cluster demarked by PC1 coordinates (-10, 10) and PC2 coordinates (−30, −15) contained 87% of the fresh market accessions from Florida and 65% of the accessions from North Carolina (Figure 1B). In contrast, the cluster from PC1 (10, 30) and PC2 (-15, 10) contained 100% of the fresh market accessions from Oregon and California. The Oregon and California accessions were not part of our previous collection [9], and the sub-structure identified in this analysis suggests that diversifying forces may play a role in shaping fresh market as well as processing tomato germplasm. Genetic differentiation within market classes may reflect founder effects in breeding programs, selection for specific traits, environmental adaptation, or a combination of these factors. Cultivated cherry, wild cherry and landrace accessions were not well separated. These results support previous studies showing that Latin American accessions and feral accessions often share alleles [69]. The lack of differentiation between cultivated cherry and wild cherry or landrace accessions was somewhat surprising, but is consistent with a diverse breeding base for the cherry market class.

Genetic diversity for each sub-population was measured using allelic richness, expected heterozygosity, and polymorphic information content. The descriptive statistics in the contemporary sub-populations exceeded levels found in the vintage sub-population, but were lower relative to the cherry sub-populations and *S. pimpinellifolium*. Although differences between processing and vintage sub-populations may be population size dependent, rarefaction analysis provides strong support for increased variation in fresh-market sub-population relative to vintage sub-population. Tomato has undergone genetic bottlenecks during domestication and through selection after the introduction of the crop into Europe [2], [70]. Since the early 1900s, wild relatives have been used to introgress new alleles into cultivated tomato [3]–[5]. This practice is expected to increase allelic diversity in contemporary processing and large-fruited fresh-market germplasm.

The analysis of minor allele frequency (MAF) across the genome demonstrates genetic differentiation between sub-populations in specific chromosome regions. The different MAF patterns between three cultivated sub-populations (processing, fresh market, and vintage) were particularly evident on chromosomes 2, 4, 5, 6, and 11. The same regions of the genome were highlighted by SNPs contributing high absolute values to PCA loadings and by SNPs detected as *F*_st_ outliers based on a deviation from the expected distribution of *F*_st_ and *He*. Thus, these chromosomes appear to be under diversifying selection relative to other regions of the genome.

In order to investigate potential regions of the genome under selection, we superimposed MFA patterns, PCA loadings, and SNPs identified from *F*_st_ outlier detection with the position of genes affecting fruit shape, size and color, disease resistance, and plant morphology (Figure 3 and Figure 4). Taken together, several regions of the genome containing candidate genes which may be under selection were detected based on coincident MAF patterns, PC loadings, or *F*_st_ outlier analysis. However, the resolution of this analysis does not provide unequivocal evidence that selection for these candidates explains variation between sub-populations. Given our marker resolution and observed LD decay, candidate gene analysis was not highly informative. In addition, our ability to detect outliers may be influenced by allele frequencies and distribution across the sub-populations. In general, several regions under selection are compatible with the introgression of genes for fruit size and shape (e.g. *lc* and *FAS*), fruit color (e.g. *gf* and *u*), disease resistance (e.g.*Cf-2* and *I-2*), and plant morphology (e.g. *s* and *SP5*).

Our results also suggest several chromosomes or regions of chromosomes with no obvious candidate genes to explain the observed differentiation. Chromosome 4 was identified as highly important for genetic differentiation between the sub-populations of cultivated germplasm. The role of this chromosome is less clear, though multiple loci affecting plant habit (e.g. *dmt, Epi, glo,* and *si*) have been mapped relative to morphological markers on this chromosome (http://tgrc.ucdavis.edu). Other areas under selection that are poorly explained by candidate genes include the chromosome 5 centromere which differentiates processing germplasm from the other sub-populations.

Common alleles at many loci in the *S. pimpinellifolium* accessions are minor alleles in the other sub-populations, including cherry tomato. A recent study with 144 cherry accessions demonstrated a separation into two groups: one close to the cultivated tomato and one showing admixture of cultivated tomato and *S. pimpinellifolium* [71]. Genetic diversity was higher in the *S. pimpinellifolium* relative to the wild cherry and cultivated tomato accessions, a result that is consistent with our findings. We expect accessions of *S. pimpinellifolium* to be relatively diverse due to their range of mating systems (many accessions exhibit facultative outcrossing) and wide geographic distribution in the native region [72]. The wild cherry accessions may be a mixture of feral cultivated accessions and wild progenitors of cultivated tomato [71].

The decay of LD over genetic distance is important to determine the density of markers appropriate for genetic analysis and selection strategies. LD levels vary both within and between species [73]. Previous estimates of LD decay in tomato were based on the entire genome with an average of 6–14 cM in processing accessions [74], 3–16 cM in fresh market accessions [74], and 15–20 cM in commercial European greenhouse accessions [75]. Given the marker density across each chromosome, we estimated LD decay on a chromosome by chromosome basis for processing, fresh market, and vintage accessions. LD decay was variable between chromosomes and sub-populations, suggesting that historical recombination is not uniform across the genome. For example, chromosome 11 showed decay over 0.8 cM for the processing sub-population, and decay over 19.7 cM for the fresh market sub-population. Although the similar estimates of LD decay was found between the LOESS and NLR methods on most of chromosomes, high levels of difference were found on chromosomes 6, 8, or 11 depending on the sub-populations. These chromosomes also showed high levels of non-homogenous distributions for pairwise *r^2^* values relative to the other chromosomes. This variation may reflect structure within sub-populations due to selection. When the *a priori* vintage germplasm sub-population was based on age of variety, regardless of fruit size or geographical origin, the LD decay pattern for chromosome 4 displayed extensive LD and what appeared to be parallel patterns of decay. When the vintage accessions were separated into large fruited vintage and cultivated cherry/landrace groups, the dual pattern of LD decay disappeared and LD was reduced (Figure S1).

The patterns of LD decay we observed appear to be a consequence of introgression and directional selection through breeding. The range of LD decay also suggests that recombination remains limiting in cultivated tomato because of the inbreeding mating system and an emphasis on backcrossing and pedigree selection in tomato breeding programs [22], [76], [77]. With LD decay exceeding 1cM, the SolCAP tomato array will be useful for most selection strategies and association studies as the average marker intervals range from 0.8 to 1.6 cM in the tomato genetic maps [19].

High-throughput SNP genotyping has provided a means to both visualize and quantify the effect of human selection on the tomato genome. Selection has reduced genetic diversity in vintage cultivated forms relative to wild and feral forms, and has changed the frequency of predominant alleles. In contrast to domestication, contemporary breeding has increased allelic diversity and heterozygosity in populations relative to vintage tomatoes. Selection has also led to sub-populations which are characterized by distinct haplotype blocks, patterns of allelic diversity, and recombination history. This analysis has highlighted specific regions of the genome that appear to be under selection, with SNPs on chromosomes 2, 4, 5, 6, and 11 clearly distinguishing fresh market and processing lineages. Our findings are not surprising given the history of breeding activities. However, incorporating this information into future breeding strategies offers both challenges and potential for creativity. Going forward, we will want to balance a desire to promote recombination and generate new combinations of alleles, with the need to preserve desirable combinations of genes. These analyses also highlight a role for several chromosomes in the differentiation of cultivated tomatoes. A role for chromosome 4 has not been highlighted by previous studies yet this chromosome appears to have regions that have been selected during breeding activities.

## Materials and Methods

### Plant Material

A collection of 426 tomato accessions, referred to as the Solanaceae Coordinated Agricultural Project (SolCAP) germplasm panel, was assembled from the National Plant Germplasm System (NPGS), the C. M. Rick Tomato Genetics Resource Center (TGRC), and from public plant breeding programs. Accessions from eight universities in the United States and Canada were represented including Cornell University (USA), North Carolina State University (USA), Ohio State University (USA), Oregon State University (USA), Pennsylvania State University (USA), University of Florida (USA), University of California-Davis (USA), and Ridgetown College, University of Guelph (Canada). The germplasm panel represented only the red-fruited species *S. lycopersicum* and *S. pimpinellifolium*, and included 141 processing, 110 fresh market, 61 vintage, 27 cultivated cherry, 12 landrace, 43 wild cherry, 16 *S. pimpinellifolium*, and 16 hybrid accessions (Table S1). Processing and fresh market germplasm represented contemporary accessions, while vintage accessions (sometime referred to as heirlooms) represented early tomato selections that in some cases predate the application of Mendelian principles to crop improvement [7], [9]. Cherry tomatoes, often referred to as *S. lycopersicum ‘cerasiforme’*, in our germplasm panel were separated into two sub-populations: cultivated and wild accessions. Landraces included Latin American cultivars and represent early domesticates from regions near the centers of origin and domestication. The collection also contained germplasm that was considered commercially relevant, with several inbred lines that are parents of commercial hybrids [78]–[80]. The collection also included the parents of several important recombinant inbred and inbred backcross populations [45], [81]–[84], segmental substitution lines [85], and a mutation library [86]. Finally, two accessions that have been the subject of public sequencing efforts, Heinz 1706 and LA1589, were also included.

### SNP Genotyping

The SolCAP germplasm panel was genotyped using a tomato array with 7,720 SNPs as implemented in the Infinium assay (Illumina Inc., San Diego, CA, USA). Details of the SolCAP SNP discovery pipeline are described previously [10], as are details of the array [19]. In addition, all SNPs on the array have been incorporated into the SGN database [87], the SNP annotation file is available through the SolCAP website [88], and sequences are available through the National Center for Biotechnology Information Sequence Read Archive (accession number SRP007969).

For each accession of the SolCAP germplasm, genomic DNA was isolated from fresh young leaf tissue according to a modified CTAB method [82]. Double-stranded DNA concentrations were quantified using the PicoGreen assay (Life Technologies Corp., Grand Island, NY, USA) and normalized to 50 ng/ul with 10 mM Tris-HCl pH 8.0, 1 mM EDTA. Genotyping was conducted with 250 ng of DNA per accession following the manufacturer’s protocol for the Infinium assay. For SNP calls, the resulting intensity data were loaded in GenomeStudio version 1.7.4 (Illumina Inc., San Diego, CA, USA). In order to determine SNP genotype, we first used the automated cluster algorithm to generate initial calls. Clustering for every SNP was assessed by visual inspection and modified when the default clustering was not clearly defined. Particular attention was paid to a clear definition of the boundaries for heterozygote calls, which were reduced manually for a number of SNPs in order to reduce the number of ambiguous calls. As a result, the rate of alleles with no call was increased slightly.

### Data Analysis

Physical positions of 7,666 SNPs were previously determined relative to the tomato genome sequence [19]. The SNPs with physical positions were filtered based on polymorphism and missing data. The 7,323 polymorphic SNPs with <10% missing data included 13 markers with inconsistent chromosome assignments between physical and genetic map positions. We removed these SNPs and used the 7,310 SNPs for minor allele frequency (MAF) and rarefaction analyses. For principal component analysis (PCA), pairwise *F*_st_, descriptive statistics, and *F*_st_ outlier detection, we used 4,393 polymorphic SNPs (excluding the 13 markers) with <10% missing data that were genetically mapped in the Moneymaker x LA0121 F_2_ population of 184 plants [19]. A second set of 3,473 markers was selected based on genetic position in the EXPEN 2000 genetic map [19] and used for pairwise estimation of *F*_st_. Polymorphism of these markers was determined based on the observation of at least one alternative allele in the 410 inbred accessions. For linkage disequilibrium (LD) analysis, the 4,393 SNP markers were filtered for a MAF of ≥10% within each sub-population.

#### Principal component analysis

The genetic relationship between sub-populations was analyzed using PCA as implemented in R [89]. GenomeStudio SNP data were converted to proportional scoring where 2 is equal to homozygous for the common allele; 1 is equal to heterozygote; and 0 is homozygous for the rare allele. Missing data were imputed using the R package pcaMethods [90] in which missing data calls are based on the SVDimpute algorithm [91]. PCA was conducted for the entire data set, and subsequently for a data set consisting of only the three major sub-populations of cultivated tomato (processing, fresh market, and vintage). The relationship between accessions was visualized by plotting scores for PCs. Marker contributions to the loadings of each PC were extracted and displayed relative to chromosome position in order to visualize regions of the genome containing markers that maximize variation within the germplasm collection and assuming that these represent regions of the genome with maximum diversity. Significant differences between sub-populations were tested via analysis of variance (ANOVA) for the eigenvalues of the first two principal components.

#### Genetic differentiation and diversity

As a measure of population differentiation, pairwise *F*_st_ [92] was estimated using the Microsatellite analyzer v4.05 [93]. This analysis was conducted using two sets of markers (3,473 and 4,393 SNPs) for all 410 inbred accessions. We also estimated pairwise *F*_st_ with a reduction of sample size (n = 40) for processing, fresh market, vintage sub-populations in order to estimate *F*_st_ without bias due to different population sizes. The analysis was iterated for three separate sets of accessions with n = 40 for each market class. The *P*-value for the pairwise *F*_st_ was obtained from 10,000 permutations of genotypes and a Bonferroni correction was applied. Genetic diversity within each sub-population was assessed based on allelic richness (*A*) [94], [95], expected heterozygosity (*He*) [96] and polymorphic information content (PIC) [97] using the 4,393 SNPs for the 410 accessions. *A* and *He* were estimated using the MSA software which corrects for sample size [98], while PIC was calculated using the equation:
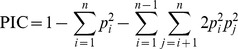
where n is the number of allele and *p_i_* is the frequency of the *i* ^th^ allele [97].

#### Rarefaction analysis

This analysis was used to estimate how the number of polymorphic markers increases relative to sample size [26]. Analysis and graphing was conducted using the species accumulation curve function “specaccum” in the R package, vegan [99]. The method “random” was applied to estimate means and standard deviations from 100 sub-samples without replacement [26]. This method provides a data summary and permits boxplot methods to be used to graph the curves. The boxplot function was used to superimpose standard deviations.

#### Minor allele frequency

The minor allele was determined based on the allele calls for 410 inbred accessions. Minor allele frequency (MAF) was then calculated across all chromosomes for each of sub-populations and sub-groups within the processing and fresh market sub-populations. This approach permitted the comparison of allele frequencies between sub-populations. MAF was graphed to visualize genetic variation based on physical map position across 12 chromosomes using the R package ggplot2 [100]. We also positioned genes for disease resistance (13 genes), fruit shape and size (5 genes), fruit color (5 genes), and plant morphology (5 genes) on the MAF plot (Table S9). Physical positions of the genes relative to the tomato genome sequences were determined using coding sequences or flanking marker sequences. These sequences were aligned to the Tomato WGS chromosome v SL2.40 [101], [102] using BLAST [103].

#### Loci under positive selection

In order to identify loci under positive selection between processing, fresh market, and vintage sub-populations, we used an *F*_st_- outlier detection method as implemented in the LOSITAN workbench [27], [28]. Outliers were detected based on a distribution of *F*_st_ and expected heterozygosity (*He*). Five simulations for each pairwise comparison were run with 10,000 iterations. Simulations were conducted using the neutral mean, forced mean, and a mutation model with infinite alleles. Under these options, LOSITAN estimated a 95% confidence interval, and SNPs that fell outside of this range were identified as under positive selection (higher than expected *F*_st_ for an estimated *He*).

#### Linkage disequilibrium (LD) analysis

The extent of LD across each chromosome was measured using the SNP genotypes in three representative groups of cultivated tomatoes (processing, fresh market, and vintage). Pairwise *r^2^* between markers within each chromosome was then calculated using TASSEL v 2.1 [104] and GGT v 2 [105]. In order to determine the decay of LD, these *r^2^* values were plotted against the genetic distance (cM) between markers. Curves of LD decay were fitted using locally weighted scatterplot smoothing (LOESS) [66] and non-linear regression (NLR) [67] using R [89]. For LOESS, smoothing parameters between 0.1 and 0.5 were tested, and a final parameter of 0.3 was chosen based on curve fits. For NLR, the predicted *r^2^* values between adjacent markers were calculated using the model of Hill and Weir [106]. Two methods were chosen to determine baseline *r^2^* values: a fixed value of 0.2 [107] and the parametric 95^th^ percentile of the distribution of the unlinked markers [108].

## Supporting Information

Figure S1 Linkage disequilibrium (LD) decay on chromosome 4 for vintage, cultivated cherry, and landrace accessions. LD measures r2 against genetic map distance between pairs of SNP markers. Decay curves are represented by red (LOESS) and blue (non-linear regression). The baseline r2values were indicated by horizontal dashed line (the 95th percentile r value of 0.11) and solid line (the fixed r2 value of 0.2).
(TIF)

Figure S2 Minor allele frequency (MAF) for further divisions within both processing and fresh market germplasm. The minor allele was determined relative to allele calls for all 410 inbred accessions based on 7,310 SNPs, and then MAF was estimated and graphed within each sub-group. Proc 1 and Proc 2 indicate sub-groups of processing germplasm, and FM 1 and FM2 indicate sub-groups of fresh market germplasm.
(TIF)

Table S1 The SolCAP germplasm panel used in this study.
(XLSX)

Table S2 SNP quality, polymorphism, map position and genotypes for the SolCAP germplasm.
(XLSX)

Table S3 Sub-groups within the processing and fresh market germplasm based on PC1 and PC2 of PCA.
(XLSX)

Table S4 Minor allele frequency of 7,310 SNP markers with physical map positions in the sub-populations and sub-groups of the SolCAP germplasm.
(XLSX)

Table S5 PCA loadings for PC1 and PC2 in the three representative sub-populations of cultivated germplasm (processing, fresh market, and vintage).
(XLSX)

Table S6 Candidates for loci under positive selection between processing and fresh market sub-populations.
(XLSX)

Table S7 Candidates for loci under positive selection between processing and vintage sub-populations.
(XLSX)

Table S8 Candidates for loci under positive selection between fresh market and vintage sub-populations.
(XLSX)

Table S9 28 tomato loci for integration with MAF patterns, PCA loadings, and *F*_st_ outlier detection.
(XLSX)

## References

[1] Tanksley SD, McCouch SR (1997) Seed banks and molecular maps: Unlocking genetic potential from the wild. Science 277: 1063–1066. 9262467 10.1126/science.277.5329.1063

[2] Miller JC, Tanksley SD (1990) RFLP analysis of phylogenetic relationships and genetic variation in the genus *Lycopersicon* . Theor Appl Genet 80: 437–448. 24221000 10.1007/BF00226743

[3] United States Department of Agriculture, States Relations Service, Office of Experiment Stations (1919) Experiment Station Record Vol 39. UNT Digital Library. Available: http://digital.library.unt.edu/ark:/67531/metadc5015/. Accessed 2012 Aug 27.

[4] Alexander LJ (1934) Leaf mold resistance in the tomato. Ohio Agri Exp Sta Bull 539.

[5] Watts VM (1947) The use of *Lycopersicon peruvianum* as a source of nematode resistance in tomatoes. Proc Amer Soc Hort Sci 49: 233–234.

[6] Park YH, West MAL, St Clair DA (2004) Evaluation of AFLPs for germplasm fingerprinting and assessment of genetic diversity in cultivars of tomato (*Lycopersicon esculentum* L.). Genome 47: 510–518. 15190368 10.1139/g04-004

[7] Williams CE, St. Clair DA (1993) Phenetic relationships and levels of variability detected by restriction fragment length polymorphism and random amplified polymorphic DNA analysis of cultivated and wild accessions of *Lycopersicon esculentum* . Genome 36: 619–630. 18470012 10.1139/g93-083

[8] Sim SC, Robbins MD, Chilcott C, Zhu T, Francis DM (2009) Oligonucleotide array discovery of polymorphisms in cultivated tomato (*Solanum lycopersicum* L.) reveals patterns of SNP variation associated with breeding. BMC Genomics 10: 10. 19818135 10.1186/1471-2164-10-466 PMC2763011

[9] Sim SC, Robbins MD, Van Deynze A, Michel AP, Francis DM (2011) Population structure and genetic differentiation associated with breeding history and selection in tomato (*Solanum lycopersicum* L.). Heredity 106: 927–935. 21081965 10.1038/hdy.2010.139 PMC3186243

[10] Hamilton JP, Sim S, Stoffel K, Van Deynze A, Buell CR, et al (2012) Single nucleotide polymorphism discovery in cultivated tomato via sequencing by synthesis. The Plant Genome 5: 17–29.

[11] Gupta PK, Rustgi S, Mir RR (2008) Array-based high-throughput DNA markers for crop improvement. Heredity 101: 5–18. 18461083 10.1038/hdy.2008.35

[12] Yang W, Bai XD, Kabelka E, Eaton C, Kamoun S, et al (2004) Discovery of single nucleotide polymorphisms in *Lycopersicon esculentum* by computer aided analysis of expressed sequence tags. Mol Breeding 14: 21–34.

[13] Labate JA, Baldo AM (2005) Tomato SNP discovery by EST mining and resequencing. Mol Breeding 16: 343–349.

[14] Jiménez-Gómez J, Maloof J (2009) Sequence diversity in three tomato species: SNPs, markers, and molecular evolution. BMC Plant Biol 9: 85. 19575805 10.1186/1471-2229-9-85 PMC3224693

[15] Van Deynze A, Stoffel K, Buell CR, Kozik A, Liu J, et al (2007) Diversity in conserved genes in tomato. BMC Genomics 8: 465. 18088428 10.1186/1471-2164-8-465 PMC2249608

[16] Labate JA, Robertson LD, Wu FN, Tanksley SD, Baldo AM (2009) EST, COSII, and arbitrary gene markers give similar estimates of nucleotide diversity in cultivated tomato (*Solanum lycopersicum* L.). Theor Appl Genet 118: 1005–1014. 19153710 10.1007/s00122-008-0957-2

[17] Steemers FJ, Chang WH, Lee G, Barker DL, Shen R, et al (2006) Whole-genome genotyping with the single-base extension assay. Nat Methods 3: 31–33. 16369550 10.1038/nmeth842

[18] Ganal MW, Durstewitz G, Polley A, Berard A, Buckler ES, et al (2011) A large maize (*Zea mays* L.) SNP genotyping array: development and germplasm genotyping, and genetic mapping to compare with the B73 reference genome. PLoS ONE 6: e28334. 22174790 10.1371/journal.pone.0028334 PMC3234264

[19] Sim S, Durstewitz G, Plieske J, Wieseke R, Ganal M, et al (2012) Development of a large SNP genotyping array and generation of high-density genetic maps in tomato. PLoS ONE 7: e40563. 22802968 10.1371/journal.pone.0040563 PMC3393668

[20] Zhao K, Tung CW, Eizenga GC, Wright MH, Ali ML, et al (2011) Genome-wide association mapping reveals a rich genetic architecture of complex traits in *Oryza sativa* . Nat Commun 2: 467. 21915109 10.1038/ncomms1467 PMC3195253

[21] Thomson MJ, Zhao K, Wright M, McNally KL, Rey J, et al (2012) High-throughput single nucleotide polymorphism genotyping for breeding applications in rice using the BeadXpress platform. Mol Breeding 29: 875–886.

[22] Graham TO (1959) Impact of recorded mendelian factors on the tomato 1929–1959. Tom Gen Coop Rep 9: 37.

[23] Rasmussen WD (1968) Advances in American agriculture: The mechanical tomato harvester as a case study. Technol Cult 9: 531–543.

[24] Sim SC, Merk HL, McQueen J (2011) Downstream analysis of SNPs from the SolCAP tomato infinium aray (II). Available: http://www.extension.org/pages/61007. Accessed 2012 Aug 27.

[25] Labate JA, Francis D, McGrath MT, Panthee D, Robertson LD (2010) Diversity in a collection of heirloom tomato varieties. HortScience 45: S145–S145.

[26] Gotelli NJ, Colwell RK (2001) Quantifying biodiversity: procedures and pitfalls in the measurement and comparison of species richness. Ecol Lett 4: 379–391.

[27] Antao T, Lopes A, Lopes RJ, Beja-Pereira A, Luikart G (2008) LOSITAN: A workbench to detect molecular adaptation based on a Fst-outlier method. BMC Bioinformatics 9: 323. 18662398 10.1186/1471-2105-9-323 PMC2515854

[28] Beaumont MA, Nichols RA (1996) Evaluating loci for use in the genetic analysis of population structure. Proc R Soc B 263: 1619–1626.

[29] Frary A, Nesbitt TC, Frary A, Grandillo S, van der Knaap E, et al (2000) *fw2.2*: a quantitative trait locus key to the evolution of tomato fruit size. Science 289: 85–88. 10884229 10.1126/science.289.5476.85

[30] Van der Hoeven R, Ronning C, Giovannoni J, Martin G, Tanksley S (2002) Deductions about the number, organization, and evolution of genes in the tomato genome based on analysis of a large expressed sequence tag collection and selective genomic sequencing. Plant Cell 14: 1441–1456. 12119366 10.1105/tpc.010478 PMC150698

[31] Liu JP, Van Eck J, Cong B, Tanksley SD (2002) A new class of regulatory genes underlying the cause of pear-shaped tomato fruit. Proc Natl Acad Sci U S A 99: 13302–13306. 12242331 10.1073/pnas.162485999 PMC130628

[32] Munos S, Ranc N, Botton E, Berard A, Rolland S, et al (2011) Increase in tomato locule number is controlled by two single-nucleotide polymorphisms located near WUSCHEL. Plant Physiol 156: 2244–2254. 21673133 10.1104/pp.111.173997 PMC3149950

[33] Xiao H, Jiang N, Schaffner E, Stockinger EJ, van der Knaap E (2008) A retrotransposon-mediated gene duplication underlies morphological variation of tomato fruit. Science 319: 1527–1530. 18339939 10.1126/science.1153040

[34] Cong B, Barrero LS, Tanksley SD (2008) Regulatory change in YABBY-like transcription factor led to evolution of extreme fruit size during tomato domestication. Nat Genet 40: 800–804. 18469814 10.1038/ng.144

[35] Solanaceae Coordinated Agricultural Project website. Available: http://solcap.msu.edu/tomato_phenotype_data.shtml. Accessed 2012 Aug 27.

[36] Sol Genomics Network website. Availalbe: http://solgenomics.net/search/phenotypes/stock. Accessed 2012 Aug 27.

[37] Merk HL, Yarnes SC, Van Deynze A, Tong N, Menda N, et al.. (2012) Trait diversity and potential for selection indices based on variation among regionally adapted processing tomato germplasm. J Amer Soc Hort Sci. In press.

[38] Luo J, Butelli E, Hill L, Parr A, Niggeweg R, et al (2008) AtMYB12 regulates caffeoyl quinic acid and flavonol synthesis in tomato: expression in fruit results in very high levels of both types of polyphenol. Plant J 56: 316–326. 18643978 10.1111/j.1365-313X.2008.03597.x

[39] Ray J, Moureau P, Bird C, Bird A, Grierson D, et al (1992) Cloning and characterization of a gene involved in phytoene synthesis from tomato. Plant Mol Biol 19: 401–404. 1623189 10.1007/BF00023387

[40] Yuan DJ, Chen J, Shen HL, Yang WC (2008) Genetics of flesh color and nucleotide sequence analysis of phytoene synthase gene 1 in a yellow-fruited tomato accession PI114490. Sci Hortic-Amsterdam 118: 20–24.

[41] Ronen G, Carmel-Goren L, Zamir D, Hirschberg J (2000) An alternative pathway to beta-carotene formation in plant chromoplasts discovered by map-based cloning of Beta and old-gold color mutations in tomato. Proc Natl Acad Sci U S A 97: 11102–11107. 10995464 10.1073/pnas.190177497 PMC27155

[42] Barry CS, McQuinn RP, Chung MY, Besuden A, Giovannoni JJ (2008) Amino acid substitutions in homologs of the STAY-GREEN protein are responsible for the green-flesh and chlorophyll retainer mutations of tomato and pepper. Plant Physiol 147: 179–187. 18359841 10.1104/pp.108.118430 PMC2330295

[43] Powell A, Nguyen C, Hill T, Cheng K, Figueroa-Balderas R, et al (2012) Uniform ripening encodes a Golden 2-like transcription factor regulating tomato fruit chloroplast development. Science 336: 1711–1715. 22745430 10.1126/science.1222218

[44] Martin GB, Brommonschenkel SH, Chunwongse J, Frary A, Ganal MW, et al (1993) Map-based cloning of a protein kinase gene conferring disease resistance in tomato. Science 262: 1432–1436. 7902614 10.1126/science.7902614

[45] Yang W, Sacks EJ, Ivey MLL, Miller SA, Francis DM (2005) Resistance in *Lycopersicon esculentum* intraspeciflc crosses to race T1 strains of *Xanthomonas campestris* pv. *vesicatoria* causing bacterial spot of tomato. Phytopathology 95: 519–527. 18943317 10.1094/PHYTO-95-0519

[46] Dixon MS, Jones DA, Keddie JS, Thomas CM, Harrison K, et al (1996) The tomato *Cf-2* disease resistance locus comprises two functional genes encoding leucine-rich repeat proteins. Cell 84: 451–459. 8608599 10.1016/s0092-8674(00)81290-8

[47] Milligan SB, Bodeau J, Yaghoobi J, Kaloshian I, Zabel P, et al (1998) The root knot nematode resistance gene *Mi* from tomato is a member of the leucine zipper, nucleotide binding, leucine-rich repeat family of plant genes. Plant Cell 10: 1307–1319. 9707531 10.1105/tpc.10.8.1307 PMC144378

[48] Scott JW, Agrama HA, Jones JP (2004) RFLP-based analysis of recombination among resistance genes to Fusarium wilt races 1, 2, and 3 in tomato. J Amer Soc Hort Sci 129: 394–400.

[49] Bohn GW, Tucker CM (1939) Immunity to fusarium wilt in the tomato. Science 89: 603–604. 17751616 10.1126/science.89.2322.603

[50] Pei CC, Wang H, Zhang JY, Wang YY, Francis DM, et al (2012) Fine mapping and analysis of a candidate gene in tomato accession PI128216 conferring hypersensitive resistance to bacterial spot race T3. Theor Appl Genet 124: 533–542. 22038434 10.1007/s00122-011-1726-1

[51] Simons G, Groenendijk J, Wijbrandi J, Reijans M, Groenen J, et al (1998) Dissection of the Fusarium *I2* gene cluster in tomato reveals six homologs and one active gene copy. Plant Cell 10: 1055–1068. 9634592 10.1105/tpc.10.6.1055 PMC144031

[52] Hemming MN, Basuki S, McGrath DJ, Carroll BJ, Jones DA (2004) Fine mapping of the tomato *I-3* gene for fusarium wilt resistance and elimination of a co-segregating resistance gene analogue as a candidate for *I-3* . Theor Appl Genet 109: 409–418. 15045176 10.1007/s00122-004-1646-4

[53] Lanfermeijer FC, Dijkhuis J, Sturre MJG, de Haan P, Hille J (2003) Cloning and characterization of the durable tomato mosaic virus resistance gene *Tm-2(2)* from *Lycopersicon esculentum* . Plant Mol Biol 52: 1037–1049. 14558663 10.1023/a:1025434519282

[54] Chunwongse J, Chunwongse C, Black L, Hanson P (2002) Molecular mapping of the *Ph-3* gene for late blight resistance in tomato. J Hortic Sci Biotech 77: 281–286.

[55] Robbins MD, Masud MAT, Panthee DR, Gardner RG, Francis DM, et al (2010) Marker-assisted selection for coupling phase resistance to tomato spotted wilt virus and *Phytophthora infestans* (late blight) in tomato. HortScience 45: 1424–1428.

[56] Kawchuk LM, Hachey J, Lynch DR, Kulcsar F, van Rooijen G, et al (2001) Tomato *Ve* disease resistance genes encode cell surface-like receptors. Proc Natl Acad Sci U S A 98: 6511–6515. 11331751 10.1073/pnas.091114198 PMC33499

[57] Spassova MI, Prins TW, Folkertsma RT, Klein-Lankhorst RM, Hille J, et al (2001) The tomato gene *Sw5* is a member of the coiled coil, nucleotide binding, leucine-rich repeat class of plant resistance genes and confers resistance to TSWV in tobacco. Mol Breeding 7: 151–161.

[58] Moreau P, Thoquet P, Olivier J, Laterrot H, Grimsley N (1998) Genetic mapping of *Ph-2*, a single locus controlling partial resistance to *Phytophthora infestans* in tomato. Mol Plant Microbe In 11: 259–269.

[59] Kim MJ, Mutschler MA (2005) Transfer to processing tomato and characterization of late blight resistance derived from *Solanum pimpinellifolium* L. L3708. J Amer Soc Hort Sci 130: 877–884.

[60] Lippman ZB, Cohen O, Alvarez JP, Abu-Abied M, Pekker I, et al (2008) The making of a compound inflorescence in tomato and related nightshades. Plos Biol 6: e288. 19018664 10.1371/journal.pbio.0060288 PMC2586368

[61] Pnueli L, CarmelGoren L, Hareven D, Gutfinger T, Alvarez J, et al (1998) The *SELF-PRUNING* gene of tomato regulates vegetative to reproductive switching of sympodial meristems and is the ortholog of *CEN* and *TFL1* . Development 125: 1979–1989. 9570763 10.1242/dev.125.11.1979

[62] Jones CM, Rick CM, Adams D, Jernstedt J, Chetelat RT (2007) Genealogy and fine mapping of obscuravenosa, a gene affecting the distribution of chloroplasts in leaf veins, and evidence of selection during breeding of tomatoes (*Lycopersicon esculentum*; Solanaceae). Am J Bot 94: 935–947. 21636462 10.3732/ajb.94.6.935

[63] Carmel-Goren L, Liu YS, Lifschitz E, Zamir D (2003) The *SELF-PRUNING* gene family in tomato. Plant Mol Biol 52: 1215–1222. 14682620 10.1023/b:plan.0000004333.96451.11

[64] Mao L, Begum D, Chuang HW, Budiman MA, Szymkowiak EJ, et al (2000) *JOINTLESS* is a MADS-box gene controlling tomato flower abscission zone development. Nature 406: 910–913. 10972295 10.1038/35022611

[65] Budiman MA, Chang SB, Lee S, Yang TJ, Zhang HB, et al (2004) Localization of *jointless-2* gene in the centromeric region of tomato chromosome 12 based on high resolution genetic and physical mapping. Theor Appl Genet 108: 190–196. 14504748 10.1007/s00122-003-1429-3

[66] Cleveland WS (1979) Robust locally weighted regression and smoothing scatterplots. J Am Stat Assoc 74: 829–836.

[67] Bates DM, Watts DG (1988) Nonlinear regression analysis and its applications. Indianapolis: Wiley Publishing, Inc.

[68] Pritchard JK, Stephens M, Donnelly P (2000) Inference of population structure using multilocus genotype data. Genetics 155: 945–959. 10835412 10.1093/genetics/155.2.945 PMC1461096

[69] Rick CM (1958) The role of natural hybridization in the derivation of cultivated tomatoes of western South America. Economic Bot 12: 346–367.

[70] Rick CM (1976) Tomato, *Lycopersicom esculentum* (Solanaceae). In: Simmonds NW, editor. Evolution of crop plants. London, England: Longman Group. 268–273.

[71] Ranc N, Munos S, Santoni S, Causse M (2008) A clarified position for *Solanum lycopersicum* var. *cerasiforme* in the evolutionary history of tomatoes (solanaceae). BMC Plant Biol 8: 130. 19099601 10.1186/1471-2229-8-130 PMC2657798

[72] Rick CM, Fobes JF, Holle M (1977) Genetic variation in *Lycopersicon pimpinellifolium*: Evidence of evolutionary change in mating systems. Plant Syst Evol 127: 139–170.

[73] Flint-Garcia SA, Thornsberry JM, Buckler ES (2003) Structure of linkage disequilibrium in plants. Annu Rev Plant Biol 54: 357–374. 14502995 10.1146/annurev.arplant.54.031902.134907

[74] Robbins MD, Sim SC, Yang W, Van Deynze A, van der Knaap E, et al (2011) Mapping and linkage disequilibrium analysis with a genome-wide collection of SNPs that detect polymorphism in cultivated tomato. J Exp Bot 62: 1831–1845. 21193580 10.1093/jxb/erq367 PMC3060673

[75] van Berloo R, Zhu AG, Ursem R, Verbakel H, Gort G, et al (2008) Diversity and linkage disequilibrium analysis within a selected set of cultivated tomatoes. Theor Appl Genet 117: 89–101. 18389208 10.1007/s00122-008-0755-x PMC2413108

[76] George WL, Berry SA (1983) Genetics and breeding of processing tomatoes. In: Gould WA, editor. Tomato production, processing and quality evaluation. Westport: AVI Publishing Company, Inc. 48–65.

[77] Stevens MA, Rick CM (1986) Genetics and Breeding. In: Athernon JG, Rudich J, editors. The Tomato Crop A Scientific Basis for Improvement. London, England: Chapman and Hall. 35–109.

[78] Gardner RG (1992) ‘Mountain Spring’ tomato; NC 8276 and NC 84173 tomato breeding lines. HortScience 27: 1233–1234.

[79] Scott JW, Baldwin EA, Klee HJ, Brecht JK, Olson SM, et al (2008) Fla. 8153 hybrid tomato; Fla. 8059 and Fla. 7907 breeding lines. HortScience 43: 2228–2230.

[80] Berry SZ, Gould WA, Wiese KL (1991) `Ohio 8245′ processing tomato. HortScience 26: 1093.

[81] Doganlar S, Frary A, Ku H, Tanksley S (2002) Mapping quantitative trait loci in inbred backcross lines of *Lycopersicon pimpinellifolium* (LA1589). Genome 45: 1189–1202. 12502266 10.1139/g02-091

[82] Kabelka E, Franchino B, Francis DM (2002) Two loci from *Lycopersicon hirsutum* LA407 confer resistance to strains of *Clavibacter michiganensis* subsp. *michiganensis* . Phytopathology 92: 504–510. 18943024 10.1094/PHYTO.2002.92.5.504

[83] Graham EB, Frary A, Kang JJ, Jones CM, Gardner RG (2004) A recombinant inbred line mapping population derived from a *Lycopersicon esculentum* x *L. pimpinellifolium* cross. Tom Gen Coop Rep 54: 22–25.

[84] Robbins MD, Darrigues A, Sim S, Masud MAT, Francis DM (2009) Characterization of hypersensitive resistance to bacterial spot race T3 (*Xanthomonas perforans*) from tomato accession PI 128216. Phytopathology 99: 1037–1044. 19671005 10.1094/PHYTO-99-9-1037

[85] Eshed Y, Zamir D (1995) An introgression line population of *Lycopersicon pennellii* in the cultivated tomato enables the identification and fine mapping of yield-associated QTL. Genetics 141: 1147–1162. 8582620 10.1093/genetics/141.3.1147 PMC1206837

[86] Menda N, Semel Y, Peled D, Eshed Y, Zamir D (2004) *In silico* screening of a saturated mutation library of tomato. Plant J 38: 861–872. 15144386 10.1111/j.1365-313X.2004.02088.x

[87] Sol Genomics Network website. Available: http://solgenomics.net/cview. Accessed 2012 Aug 27.

[88] Solanaceae Coordinated Agricultural Project website. Available: http://solcap.msu.edu/tomato_genotype_data.shtml. Accessed 2012 Aug 27.

[89] R Development Core Team (2011) R: A Language and Environment for Statistical Computing. Vienna, Austria: R Foundation for Statistical Computing.

[90] Stacklies W, Redestig H, Scholz M, Walther D, Selbig J (2007) pcaMethods - a bioconductor package providing PCA methods for incomplete data. Bioinformatics 23: 1164–1167. 17344241 10.1093/bioinformatics/btm069

[91] Troyanskaya O, Cantor M, Sherlock G, Brown P, Hastie T, et al (2001) Missing value estimation methods for DNA microarrays. Bioinformatics 17: 520–525. 11395428 10.1093/bioinformatics/17.6.520

[92] Weir BS, Cockerham CC (1984) Estimating F-statistics for the analysis of populatoin structure. Evolution 38: 1358–1370. 28563791 10.1111/j.1558-5646.1984.tb05657.x

[93] Dieringer D, Schlotterer C (2003) MICROSATELLITE ANALYSER (MSA): a platform independent analysis tool for large microsatellite data sets. Mol Ecol Notes 3: 167–169.

[94] El Mousadik A, Petit RJ (1996) High level of genetic differentiation for allelic richness among populations of the argan tree [*Argania spinosa* (L) Skeels] endemic to Morocco. Theor Appl Genet 92: 832–839. 24166548 10.1007/BF00221895

[95] Hurlbert SH (1971) The nonconcept of species diversity: a critique and alternative parameters. Ecology 52: 577–586. 28973811 10.2307/1934145

[96] Nei M (1978) Estimation of average heterozygosity and genetic distance from a small number of individuals. Genetics 89: 583–590. 17248844 10.1093/genetics/89.3.583 PMC1213855

[97] Botstein D, White RL, Skolnick M, Davis RW (1980) Construction of a genetic linkage map in man using restriction fragment length polymorphisms. Am J Hum Genet 32: 314–331. 6247908 PMC1686077

[98] Microsatellite Analyzer User Manual. Available: http://i122server.vu-wien.ac.at/MSA/info.html/MSA_info.html. Accessed 2012 Aug 27.

[99] Oksanen J, Blanchet F, Kindt R, Legendre P, Minchin P, et al. (2011) Vegan: Community ecology package. Avaiable: http://cran.r-project.org/web/packages/vegan//index.html. Accessed 2012 Aug 27. R package version 20–2.

[100] Wickham H (2009) ggplot2: elegant graphics for data analysis. New York: Springer.

[101] International Tomato Genome Sequencing Project website. Available: http://solgenomics.net/organism/Solanum_lycopersicum/genome. Accessed 2012 Aug 27.

[102] The Tomato Genome Consortium (2012) The tomato genome sequence provides insights into fleshy fruit evolution. Nature 485: 635–641. 22660326 10.1038/nature11119 PMC3378239

[103] Sol Genomics Network website. Available: http://solgenomics.net/tools/blast/index.pl. Accessed 2012 Aug 27.

[104] Bradbury PJ, Zhang Z, Kroon DE, Casstevens TM, Ramdoss Y, et al (2007) TASSEL: software for association mapping of complex traits in diverse samples. Bioinformatics 23: 2633–2635. 17586829 10.1093/bioinformatics/btm308

[105] van Berloo R (2008) GGT 2.0: versatile software for visualization and analysis of genetic data. J Hered 99: 232–236. 18222930 10.1093/jhered/esm109

[106] Hill WG, Weir BS (1988) Variances and covariances of squared linkage disequilibria in finite populations. Theor Popul Biol 33: 54–78. 3376052 10.1016/0040-5809(88)90004-4

[107] Hayes BJ, Bowman PJ, Chamberlain AJ, Goddard ME (2009) Invited review: Genomic selection in dairy cattle: Progress and challenges. J Dairy Sci 92: 433–443. 19164653 10.3168/jds.2008-1646

[108] Breseghello F, Sorrells ME (2006) Association mapping of kernel size and milling quality in wheat (*Triticum aestivum* L.) cultivars. Genetics 172: 1165–1177. 16079235 10.1534/genetics.105.044586 PMC1456215

