# Supplementary figures and images for: High-Density SNP Genotyping of Tomato (Solanum lycopersicum L.) Reveals Patterns of Genetic Variation Due to Breeding
Source: PLoS One. 2012 Sep 20;7(9):e45520. doi: 10.1371/journal.pone.0045520 (PMC3447764; doi:10.1371/journal.pone.0045520)

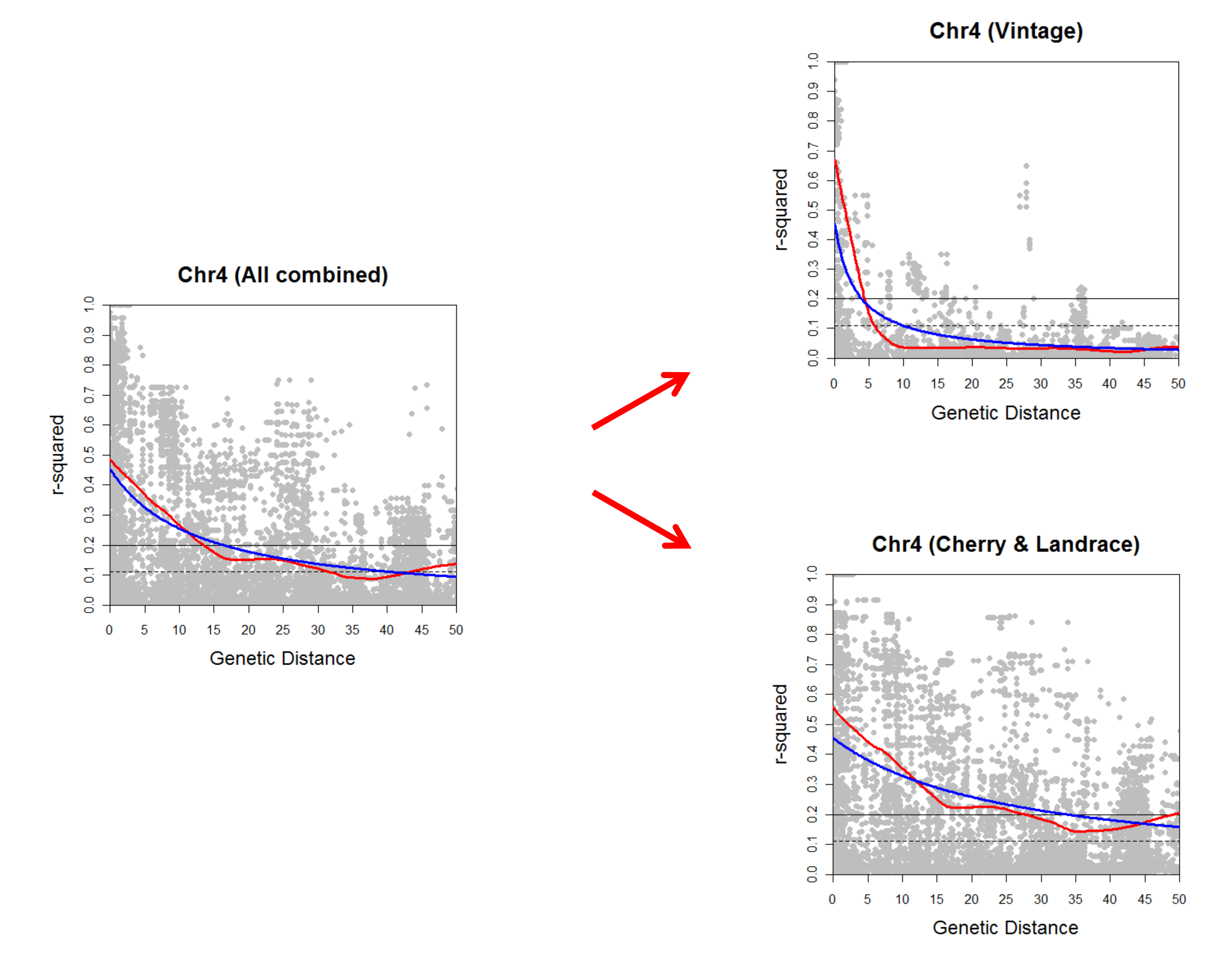

Supplement: Figure S1 — Linkage disequilibrium (LD) decay on chromosome 4 for vintage, cultivated cherry, and landrace accessions. LD measures r2 against genetic map distance between pairs of SNP markers. Decay curves are represented by red (LOESS) and blue (non-linear regression). The baseline r2values were indicated by horizontal dashed line (the 95th percentile r value of 0.11) and solid line (the fixed r2 value of 0.2). (TIF) [file pone.0045520.s001.tif]

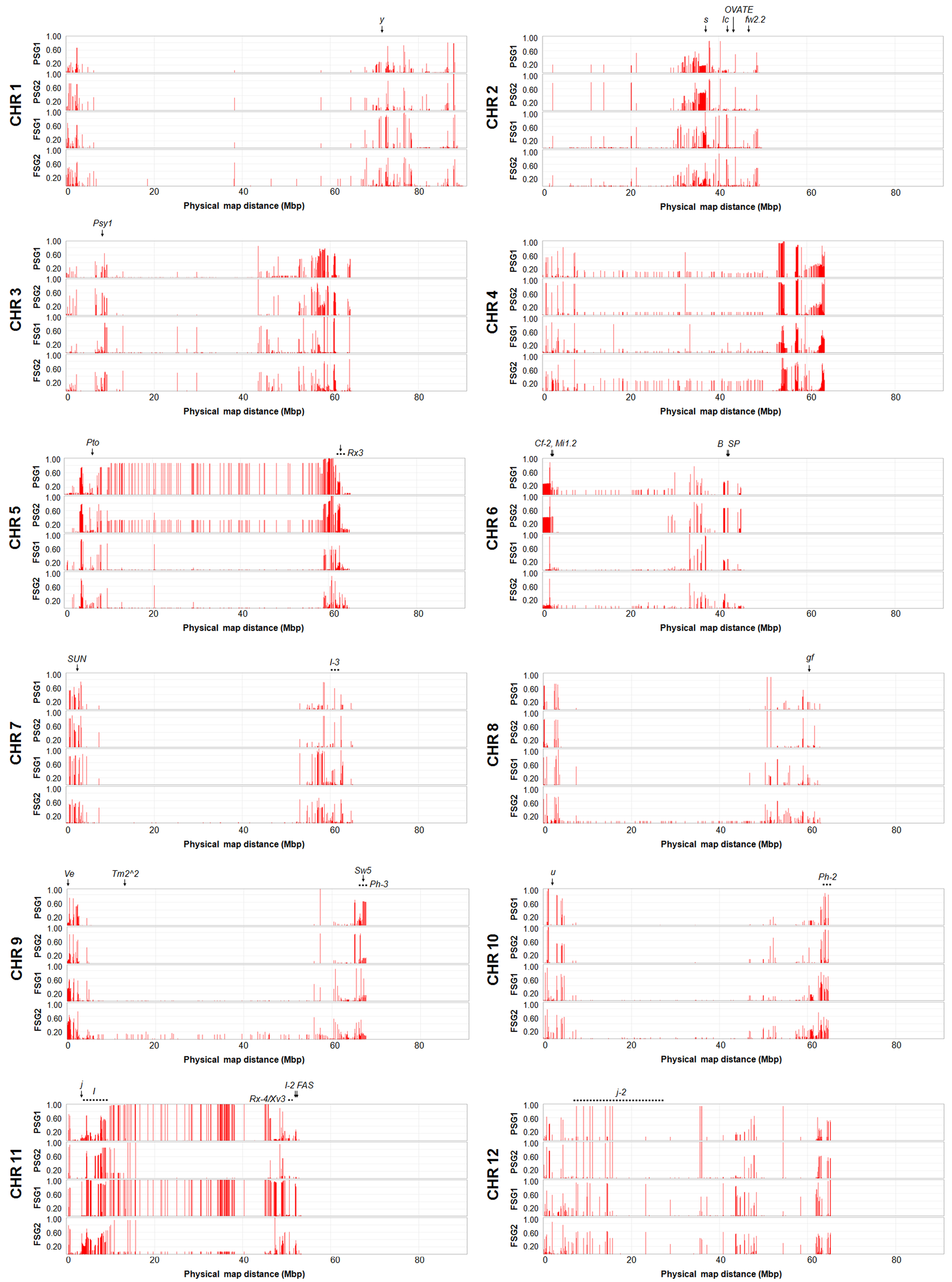

Supplement: Figure S2 — Minor allele frequency (MAF) for further divisions within both processing and fresh market germplasm. The minor allele was determined relative to allele calls for all 410 inbred accessions based on 7,310 SNPs, and then MAF was estimated and graphed within each sub-group. Proc 1 and Proc 2 indicate sub-groups of processing germplasm, and FM 1 and FM2 indicate sub-groups of fresh market germplasm. (TIF) [file pone.0045520.s002.tif]
